# Association of Circulating Tumor DNA Testing Before Tissue Diagnosis With Time to Treatment Among Patients With Suspected Advanced Lung Cancer: The ACCELERATE Nonrandomized Clinical Trial

**DOI:** 10.1001/jamanetworkopen.2023.25332

**Published:** 2023-07-25

**Authors:** Miguel García-Pardo, Kasia Czarnecka-Kujawa, Jennifer H. Law, Alexandra M. Salvarrey, Roxanne Fernandes, Zhen J. Fan, Thomas K. Waddell, Kazuhiro Yasufuku, Geoffrey Liu, Laura L. Donahoe, Andrew Pierre, Lisa W. Le, Tharsiga Gunasegaran, Noor Ghumman, Frances A. Shepherd, Penelope A. Bradbury, Adrian G. Sacher, Sabine Schmid, Lucy Corke, Jamie Feng, Tracy Stockley, Prodipto Pal, Patrik Rogalla, Christodoulos Pipinikas, Karen Howarth, Bana Ambasager, Laura Mezquita, Ming S. Tsao, Natasha B. Leighl

**Affiliations:** 1 Division of Medical Oncology and Hematology, Princess Margaret Cancer Centre, University Health Network, Toronto, Ontario, Canada; 2 Department of Medicine, University of Barcelona, Barcelona, Spain; 3 Department of Medical Oncology, Hospital Universitario Ramón y Cajal, Madrid, Spain; 4 Division of Thoracic Surgery, University Health Network, Toronto, Ontario, Canada; 5 Department of Biostatistics, Princess Margaret Cancer Centre, University Health Network, Toronto, Ontario, Canada; 6 Department of Medical Oncology, Inselspital, Bern University Hospital, University of Bern, Bern, Switzerland; 7 Pathology and Laboratory Medicine Program, University Health Network, Toronto, Ontario, Canada; 8 Department of Medical Imaging, University Health Network, Toronto, Ontario, Canada; 9 Inivata Ltd, Cambridge, United Kingdom; 10 Department of Medical Oncology, Hospital Clínic de Barcelona, Instituto de Investigaciones Biomédicas August Pi i Sunyer (IDIBAPS), Barcelona, Spain

## Abstract

**Importance:**

Liquid biopsy has emerged as a complement to tumor tissue profiling for advanced non–small cell lung cancer (NSCLC). The optimal way to integrate liquid biopsy into the diagnostic algorithm for patients with newly diagnosed advanced NSCLC remains unclear.

**Objective:**

To evaluate the use of circulating tumor DNA (ctDNA) genotyping before tissue diagnosis among patients with suspected advanced NSCLC and its association with time to treatment.

**Design, Setting, and Participants:**

This single-group nonrandomized clinical trial was conducted among 150 patients at the Princess Margaret Cancer Centre–University Health Network (Toronto, Ontario, Canada) between July 1, 2021, and November 30, 2022. Patients referred for investigation and diagnosis of lung cancer were eligible if they had radiologic evidence of advanced lung cancer prior to a tissue diagnosis.

**Interventions:**

Patients underwent plasma ctDNA testing with a next-generation sequencing (NGS) assay before lung cancer diagnosis. Diagnostic biopsy and tissue NGS were performed per standard of care.

**Main Outcome and Measures:**

The primary end point was time from referral to treatment initiation among patients with advanced nonsquamous NSCLC using ctDNA testing before diagnosis (ACCELERATE [Accelerating Lung Cancer Diagnosis Through Liquid Biopsy] cohort). This cohort was compared with a reference cohort using standard tissue genotyping after tissue diagnosis.

**Results:**

Of the 150 patients (median age at diagnosis, 68 years [range, 33-91 years]; 80 men [53%]) enrolled, 90 (60%) had advanced nonsquamous NSCLC. The median time to treatment was 39 days (IQR, 27-52 days) for the ACCELERATE cohort vs 62 days (IQR, 44-82 days) for the reference cohort (*P* < .001). Among the ACCELERATE cohort, the median turnaround time from sample collection to genotyping results was 7 days (IQR, 6-9 days) for plasma and 23 days (IQR, 18-28 days) for tissue NGS (*P* < .001). Of the 90 patients with advanced nonsquamous NSCLC, 21 (23%) started targeted therapy before tissue NGS results were available, and 11 (12%) had actionable alterations identified only through plasma testing.

**Conclusions and Relevance:**

This nonrandomized clinical trial found that the use of plasma ctDNA genotyping before tissue diagnosis among patients with suspected advanced NSCLC was associated with accelerated time to treatment compared with a reference cohort undergoing standard tissue testing.

**Trial Registration:**

ClinicalTrials.gov Identifier: NCT04863924

## Key Points

**Question:**

Can we improve time to treatment using circulating tumor DNA (ctDNA) genotyping before tissue diagnosis among patients with suspected advanced lung cancer?

**Findings:**

In this nonrandomized clinical trial, 150 patients with suspected advanced lung cancer underwent ctDNA testing during initial diagnostic workup; 90 patients had tissue confirmation of advanced nonsquamous non–small cell lung cancer. Median time to treatment was 39 days vs 62 days for a reference cohort, with faster turnaround time for genotyping results.

**Meaning:**

The use of plasma ctDNA testing before tissue diagnosis among patients with suspected advanced lung cancer may expedite biomarker testing and accelerate time to treatment.

## Teaser

This nonrandomized clinical trial evaluates the use of circulating tumor DNA genotyping before tissue diagnosis among patients with suspected advanced non–small cell lung cancer and its association with time to treatment.

## Introduction

Clinical management of newly diagnosed non–small cell lung cancer (NSCLC) requires knowledge of tumor molecular alterations to guide treatment decisions.^1,2^ Molecular testing of tumor tissue is the criterion standard for pathologic diagnosis and genotyping.^3^ However, a major barrier to personalizing cancer treatment and access to targeted therapy is incomplete or delayed tumor tissue genotyping.^4,5^ A significant proportion of patients with lung cancer do not have test results available at the time of oncology consultation,^6,7^ which can lead to prolonged wait times, worse outcomes, and defaulting to chemotherapy treatment in the absence of molecular results.^5,8,9,10^ Although multidisciplinary diagnostic assessment programs can reduce wait times for diagnosis and treatment of lung cancer,^11^ diagnostic resources, such as biopsies, are often limited, and wait times increased in many jurisdictions during the COVID-19 pandemic.^12^

Liquid biopsies are minimally invasive blood tests that identify circulating tumor DNA (ctDNA) in patients’ plasma.^13^ Use of plasma ctDNA has been shown to be noninferior to molecular genotyping of tumor tissue among patients with advanced NSCLC.^14,15^ Plasma ctDNA testing has shown clinical utility as a complementary tool in NSCLC molecular diagnosis, especially when tissue or time for molecular profiling is limited.^16,17^ Although considered a valid tool for genotyping, the optimal way to integrate liquid biopsy into the diagnostic algorithm for patients with newly diagnosed advanced NSCLC remains unclear.^18,19^ A small pilot study using a limited ctDNA next-generation sequencing (NGS) panel confirmed the feasibility of this approach, with preliminary evidence of faster NGS turnaround times and shorter time to treatment with a prediagnostic or “plasma-first” approach.^20^ More patients had actionable genomic alterations identified and were able to access targeted therapy earlier compared with a reference cohort using the standard tissue-first approach. Herein we report the clinical utility of the use of plasma ctDNA genotyping prior to a pathologic diagnosis of lung cancer using InVisionFirst-Lung (Inivata Ltd), a plasma ctDNA comprehensive NGS assay including variants, fusions, and copy number alterations relevant in lung cancer, as part of a multidisciplinary centralized referral program to expedite diagnosis and treatment of lung cancer.

## Methods

The ACCELERATE (Accelerating Lung Cancer Diagnosis Through Liquid Biopsy) study is a prospective, single-group, minimally invasive nonrandomized clinical trial conducted at the Princess Margaret Cancer Centre–University Health Network (UHN), Toronto, Ontario, Canada. Conduct of this study was approved by the institutional research ethics board (UHN Board C) and was carried out in accordance with the principles of the Declaration of Helsinki.^21^ All participants provided written informed consent prior to study enrollment. This study adhered to the Consolidated Standards of Reporting Trials (CONSORT) reporting guideline. The trial protocol is available in Supplement 1.

### Patients

Between July 1, 2021, and November 30, 2022, 150 patients were enrolled in the ACCELERATE cohort. Patients referred to the UHN Lung Rapid Assessment and Management Program (LungRAMP) were eligible to participate if they had (1) radiologic evidence of unresectable stage III or IV lung cancer; (2) measurable disease with 1 cm or more of disease detected by computed tomography; or (3) a diagnostic tissue biopsy planned or performed without a diagnosis of NSCLC yet. Eligibility was confirmed by the weekly Lung RAMP multidisciplinary case conference (MCC; including interventional respirologists, thoracic surgeons, radiologists, and medical and radiation oncologists). Patients with pleural effusions but no measurable disease were eligible if the MCC favored a diagnosis of malignant disease based on imaging. Patients were excluded if they had concurrent cancer, had a cancer diagnosis other than lung cancer in the past 2 years, or were pregnant due to potentially confounding ctDNA results. Patient self-reported race and/or ethnicity were obtained from the electronic medical record to further characterize the study population, as the frequency of molecular alterations in lung cancer can vary across different ancestral populations.^22^

### Study Procedures

**Figure 1.**
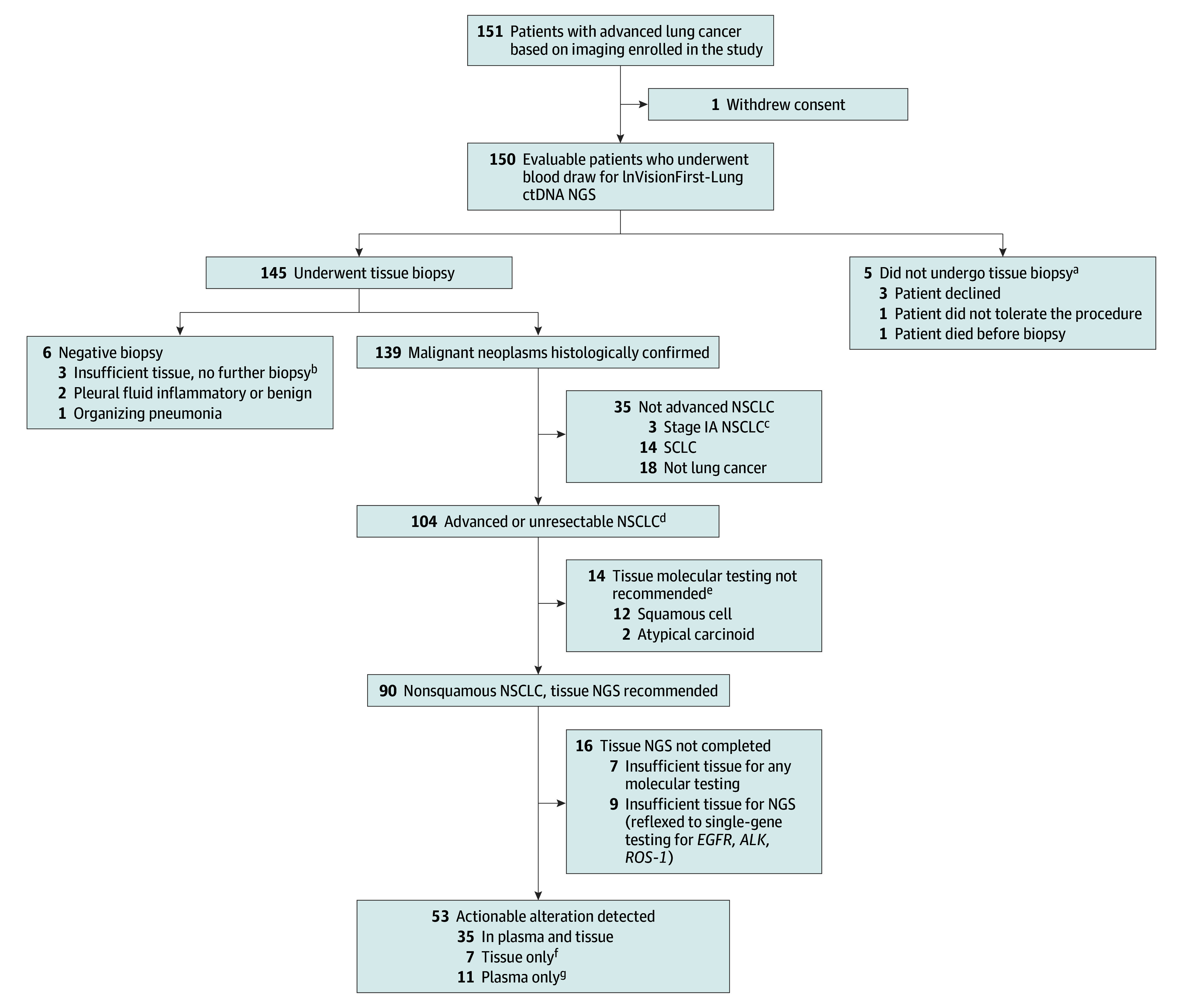
Flow Diagram of Enrolled Patients ctDNA indicates circulating tumor DNA; NGS, next-generation sequencing; NSCLC, non–small cell lung cancer; and SCLC, small cell lung cancer. ^a^Three patients with insufficient tissue for diagnosis had a plasma variant detected (*STK11*, *KRAS*^G12V^, and *PTEN*). ^b^Three patients with no biopsy had a plasma variant detected (*TP53*, *U2AF1*, and *ERBB2* [formerly *HER2* or *HER2/neu*] amplification). ^c^Three patients with stage IA NSCLC (1 benign pleural effusion, 1 benign bone lesion, and 1 benign liver cyst). ^d^Nine patients had unresectable stage III disease (6 adenocarcinoma and 3 squamous carcinoma). ^e^No patients with squamous carcinoma or atypical cardinoid histologic characteristics had informative plasma results. ^f^Of 7 patients with an actionable alteration in tissue only, 6 had undetectable ctDNA. ^g^Of 11 patients with an actionable alteration in plasma only, 6 had insufficient tissue for NGS.

After eligibility confirmation, patients were invited to participate in the study (Figure 1). Consenting patients underwent a peripheral blood draw; approximately 20 mL of blood was collected in Streck tubes and shipped to the Inivata Clinical Laboratory Improvement Amendments–accredited laboratory for analysis. Patients’ diagnostic workup continued per institutional standard, including mandatory diagnostic tumor tissue biopsy. Patients with confirmed NSCLC had reflex molecular testing per standard of care, including tissue NGS for nonsquamous NSCLC. Plasma results were returned to the study team and reviewed with the patient. Patients with actionable alterations identified in plasma received expedited medical oncology assessment. Whenever possible, treatment initiation was delayed until tumor biopsy was performed unless patients were too unwell and needed to start treatment earlier.

### Plasma ctDNA Testing

Plasma ctDNA testing was performed using InVisionFirst-Lung, a highly sensitive and specific NGS assay targeting 37 genes that has been prospectively validated and demonstrates high concordance with tissue results and high sensitivity and specificity for variants, single-nucleotide variants, fusions, and copy number alterations (eFigure 1 in Supplement 2).^23^ Blood samples were processed to plasma by centrifugation and stored at −80 °C according to validated specifications until analysis in batch. Plasma circulating free DNA (cfDNA) was extracted with the QIAamp Circulating Nucleic Acid kit (QIAGEN). After quality control, sequencing libraries were prepared using a 2-step amplification process, and libraries were sequenced by the NextSeq500 system (Illumina Inc). Sequencing data were analyzed using the Inivata analytical pipeline to identify genomic alterations. Results were returned to the study team and treating clinician within a median of 7 calendar days from blood collection.

### Tumor Tissue Molecular Testing

Reflex molecular testing of tumor tissue was performed per institutional standard of care, including comprehensive NGS (Oncomine Comprehensive Assay, version 3; Thermo Fisher Scientific) (eFigure 1 in Supplement 2) and immunohistochemistry (IHC) for tumor programmed cell death ligand 1 (PD-L1).^24,25^ Testing was initiated by pathologists on tissue NSCLC diagnosis independent of plasma results. For samples with insufficient tissue or expedited requests, single-gene testing for *EGFR* (RT-52; EntroGen Inc) and IHC for *ALK* (5A4 antibody) and *ROS-1* (D4D6 antibody, fluorescence in situ hybridization) were performed.^26,27^

For patients in the reference cohort, reflex testing using an NGS assay targeting 15 genes (Trusight Tumor 15 Panel, Illumina Inc) (eFigure 1 in Supplement 2), including *EGFR*, *BRAF*, and *KRAS*, as well as single-gene testing for *ALK* (IHC) and *ROS1* (IHC, fluorescence in situ hybridization) and PD-L1 tumor expression, were used. Further details on institutional testing methods are listed in eTable 1 in Supplement 2.

### Study End Points

The primary end point was time to treatment, defined as the time from diagnostic program referral to systemic treatment initiation for patients with advanced nonsquamous NSCLC enrolled in the ACCELERATE cohort using ctDNA genotyping prior to pathologic diagnosis compared with a reference cohort of consecutive patients referred to the program in 2018 to 2019, prior to the COVID-19 pandemic. Secondary end points included the frequency of actionable targets identified using plasma ctDNA testing, time to sample collection, and turnaround time of plasma vs tumor tissue profiling for all patients with advanced nonsquamous NSCLC. Actionable alterations were classified according to the European Society for Medical Oncology Scale for Clinical Actionability of Molecular Targets (ESCAT).^28^ Nonactionable driver alterations detected in ctDNA were considered informative tumor-derived results, assessed by OncoKB.^29^

### Reference Cohort

The LungRAMP program maintains a prospective database of all patients referred for investigation of suspected lung cancer. A reference cohort of 89 patients with advanced NSCLC diagnosed through LungRAMP and treated at the Princess Margaret Cancer Centre prior to the COVID-19 pandemic (2018-2019) was identified. Demographic characteristics, molecular data, result turnaround time, and time to treatment were abstracted.

### Statistical Analysis

Patient characteristics and molecular data are summarized using median, IQR, frequency, and percentage. The nonparametric Wilcoxon-Mann-Whitney test was used to compare time to treatment for patients with advanced nonsquamous NSCLC enrolled in the ACCELERATE cohort and the reference cohort. The Wilcoxon signed rank test was used to compare the turnaround time of plasma ctDNA testing vs tissue testing in the ACCELERATE cohort. All analyses were performed using SAS, version 9.4 (SAS Institute Inc). All *P* values were from 2-sided tests, and results were deemed statistically significant at *P* < .05.

## Results

Between July 1, 2021, and November 30, 2022, 150 patients (median age at diagnosis, 68 years [range, 33-91 years]; 80 men [53%] and 70 women [47%]; and 61 patients [41%] were never smokers) were enrolled in the ACCELERATE cohort (Figure 1 and Table). Most participants (111 [74%]) enrolled prior to diagnostic tissue biopsy and 39 (26%) enrolled after biopsy but before diagnosis. Demographic and disease characteristics of the cohort are shown in the Table. Characteristics of the reference cohort (n = 89) were similar to those in the ACCELERATE cohort.

**Table.** Baseline Characteristics and Histologic Diagnoses and Stage in the ACCELERATE and Reference Groups

Characteristic	Patients, No./total No. (%)	
	ACCELERATE cohort	Reference group
Age at diagnosis, median (range), y	68 (33-91)	68 (39-91)
Sex		
Female	70/150 (47)	47/89 (53)
Male	80/150 (53)	42/89 (47)
Smoking history		
Never smoker	61/150 (41)	32/89 (36)
Light ex-smoker (<15 packs/y)	9/150 (6)	11/89 (12)
Former	63/150 (42)	28/89 (32)
Current	17/150 (11)	18/89 (20)
ECOG performance status^a^		
0	38/150 (25)	NA
1	62/150 (41)	
2	37/150 (25)	
3	13/150 (9)	
Race and ethnicity^a^		
Asian	47/126 (37)	NA
Black	1/126 (1)	
Hispanic	5/126 (4)	
White	73/126 (58)	
Diagnosis		
Malignant neoplasm	139/150 (93)	89/89 (100)
Negative biopsy	6/150 (4)	
Not biopsied^b^	5/150 (3)	
Primary tumor		
NSCLC	107/150 (71)	89/89 (100)
Small cell lung cancer	14/150 (9)	
Not lung primary^c^	18/150 (12)	
Histologic subtypes (NSCLC)		
Adenocarcinoma	81/107 (76)	82/89 (92)
Squamous carcinoma	12/107 (11)	0
Large cell	5/107 (5)	4/89 (5)
Sarcomatoid	1/107 (1)	0
NSCLC NOS	5/107 (5)	3/89 (3)
Atypical carcinoid	2/107 (2)	0
Lymphoepithelioma-like	1/107 (1)	0
Final stage at diagnosis (NSCLC)^d^		
I-II	3/107 (3)	0
III	10/107 (9)	10/89 (11)
IV	94/107 (88)	79/89 (89)

ACCELERATE, Accelerating Lung Cancer Diagnosis Through Liquid Biopsy; ECOG, Eastern Cooperative Oncology Group; NA, not applicable; NOS, not otherwise specified; NSCLC, non–small cell lung cancer.

^a^ Not documented in the ACCELERATE cohort: ethnicity (n = 24). ECOG performance status and ethnicity were not systematically collected in the historical Lung Rapid Assessment and Management Program cohort.

^b^ A total of 5 patients did not undergo tissue biopsy: 3 declined, 1 did not tolerate procedure, and 1 underwent rapid deterioration and death before tissue biopsy was done.

^c^ A total of 18 patients with non–lung cancer diagnoses including diffuse large B-cell lymphoma (n = 4); thoracic metastases from breast, prostate, and gastrointestinal neuroendocrine carcinoma (2 each); gastrointestinal stromal tumor; Hodgkin lymphoma; carcinoma of unknown primary; melanoma; mesothelioma; plasmacytoma; endometrial adenocarcinoma; and uterine leiomyosarcoma (1 each).

^d^ A total of 3 patients with stage IA NSCLC in the ACCELERATE cohort: 1 with pleural effusion (later determined to be nonmalignant), 1 with suspicious liver lesion (later determined to be benign cysts), and 1 with suspicious bone lesion (benign bone island).

### Tissue Diagnosis

Biopsy-proven NSCLC was demonstrated in 107 patients (71%), although 3 patients were later found to have early-stage disease and underwent surgical resection (benign pleural effusion and bone and liver lesions). Of 104 patients with advanced NSCLC, 90 patients had nonsquamous histologic characteristics, 2 had atypical carcinoid tumors, and 12 had squamous carcinoma (Figure 1). Another 14 patients (9%) had small cell lung cancer and 18 (12%) had metastasis from non–lung cancer primary tumors. Five patients (3%) did not undergo biopsy; 6 (4%) had nondiagnostic biopsies with insufficient tissue or nonmalignant diagnosis. Only patients with a confirmed cancer diagnosis received systemic treatment.

### NGS Testing in Plasma and Tissue

All participants underwent plasma testing; 115 (77%) had tumor-associated variants detected (Figure 1). Of the 90 patients with advanced nonsquamous NSCLC, 46 (51%) had ESCAT tier 1 actionable driver alterations detected, 20 (22%) had nonactionable driver alterations, and 24 (27%) had uninformative results, including 16 (18%) with no detectable cfDNA. Nineteen of 20 patients with nonactionable drivers in plasma had similar results from tissue, with no additional drivers identified. These included variants in *TP53* (n = 10), *KRAS*^G12V^ (n = 6), *KRAS*^G12D^ (n = 1), *STK11* (n = 2), and *PIK3CA* (n = 1). Comparison of tissue vs ctDNA results for the guideline-recommended biomarkers in NSCLC is shown in eTable 2 in Supplement 2.

Among the 18 patients with other cancer diagnoses, 7 did not have detectable cfDNA in plasma, but 3 had potentially informative results based on cancer type (eTable 3 in Supplement 2). Three of 5 patients who did not undergo biopsy and all 3 patients with insufficient tissue from biopsy for diagnosis had tumor-associated variants detected in plasma. Of the 90 patients with advanced nonsquamous NSCLC, 74 (82%) had successful comprehensive NGS in tissue, 9 (10%) had sufficient tissue for single-gene testing only, and 7 (8%) had insufficient tissue for any testing (Figure 1); 42 patients (47%) had ESCAT tier 1 actionable alterations identified in tissue.

Combining plasma and tissue results, 53 of 90 patients (59%) had actionable alterations identified by either method: 35 of 53 (66%) in both, 11 of 53 (21%) in plasma only, and 7 of 53 (13%) in tissue only (eFigure 2 in Supplement 2). Of 7 patients with alterations in tissue only, 6 had undetectable plasma cfDNA; of 11 patients with alterations in plasma only, 6 had insufficient tissue for NGS. Except for 1 case with NSCLC not otherwise specified, actionable alterations were identified in adenocarcinoma cases. No ESCAT tier 1 to 3 variants were detected in squamous carcinoma cases.

### Time to Treatment

**Figure 2.**
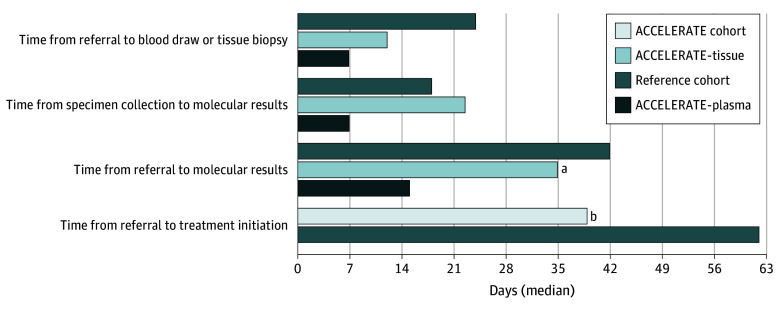
Turnaround Times ^a^A total of 74 patients had enough tissue for next-generation sequencing. ^b^A total of 72 patients started systemic therapy.

Most patients (72 of 90 [80%]) started systemic therapy for advanced nonsquamous NSCLC. Median time to treatment was 39 days (IQR, 27-52 days) for the ACCELERATE cohort compared with 62 days (IQR, 44-82 days) for the reference cohort (*P* < .001) (Figure 2).

Three-fourths (40 of 53 [75%]) of patients with actionable alterations started targeted therapy, with a median time to treatment of 33 days (IQR, 23-46 days) in the ACCELERATE cohort vs 61 days in the reference cohort (IQR, 43-80 days) (*P* < .001). Furthermore, 21 of 90 patients (23%) with advanced nonsquamous NSCLC started targeted therapy based on ctDNA findings before tissue genotyping results were available. For this subgroup, the median time to treatment was 27 days (IQR, 21-35 days). Patients who received both plasma and tissue NGS results before starting therapy had a median time to treatment of 41 days (IQR, 27-50 days).

Reasons for not starting targeted therapy among patients with actionable alterations included no approved or available first-line targeted therapy in Canada at that time (7 *KRAS* G12C, 2 *EGFR* exon 20 insertions, 1 *BRAF* V600E, and 1 *ERBB2* exon 20 insertion). One patient with *EGFR* L858R–variant lung cancer received chemoradiotherapy after multidisciplinary team review. Another patient with *MET* ex14 skip–variant lung cancer experienced rapid deterioration and died before starting treatment.

### NGS Turnaround Time and Wait Times

In the ACCELERATE cohort, the median turnaround time for plasma testing from blood draw to result reporting was 7 days (IQR, 6-9 days) vs 23 days (IQR, 18-28 days) for tissue NGS, measured from tissue biopsy to reporting (*P* < .001) (Figure 2). The time from referral to blood draw was shorter (median, 7 days [IQR, 3-11 days]) than the time from referral to tumor biopsy (median, 12 days [IQR, 6-20 days]) in the study cohort. The median time from LungRAMP referral to the plasma ctDNA result was 15 days (IQR, 4-66 days) and to the tumor tissue NGS result was 35 days (IQR, 2-88 days). Plasma NGS results were available a median of 17 days (IQR, 12-32 days) before tissue NGS results.

In the reference cohort, referred before the COVID-19 pandemic (Figure 2), the median time to tissue biopsy was longer than in the ACCELERATE cohort (24 days [IQR, 14-36 days] vs 12 days [IQR, 5-21 days]). Tissue NGS reporting turnaround times were similar, and the median time from referral to molecular result reporting in the reference cohort was 42 days (IQR, 30-55 days) compared with 35 days (IQR, 29-48 days) in the ACCELERATE cohort.

## Discussion

In our study, plasma ctDNA testing before diagnosis in the initial diagnostic workup of patients with suspected advanced NSCLC was associated with shorter time to molecular results than tissue testing and shorter median time to treatment compared with a reference cohort using tissue molecular testing only (39 vs 62 days). Plasma testing was associated with greater detection of ESCAT tier 1 actionable alterations compared with tissue testing alone. Nearly one-fourth of patients started treatment based on plasma results after tissue diagnosis and before tissue NGS results were available, with a median time to treatment of 27 days. These results suggest the clinical utility of adding plasma ctDNA testing before tissue biopsy or confirmed cancer diagnosis to the standard diagnostic workup of patients with suspected advanced lung cancer. Compared with a reference cohort using tissue molecular testing only, the use of liquid biopsy was associated with faster return of molecular results and accelerated time to treatment for all patients, both with and without targetable alterations identified.

Preliminary evidence suggests that concurrent plasma ctDNA and tissue NGS testing after lung cancer diagnosis increases the proportion of patients receiving comprehensive molecular testing vs a tissue-only approach.^30,31^ This testing leads to increased delivery of targeted therapy for patients with advanced NSCLC. To improve access and timely treatment initiation, liquid biopsy could be performed even earlier, as in our study, as part of the initial diagnosis workup for patients with suspected advanced lung cancer before tissue biopsy or diagnosis. Several recent small studies have demonstrated that plasma testing for patients with suspected advanced lung cancer at the time of or before tumor biopsy is associated with decreased time to treatment.^32,33,34^

To our knowledge, this is the largest study exploring this prediagnostic approach for suspected advanced lung cancer. Our study demonstrated that plasma ctDNA testing before lung cancer diagnosis was associated with faster time to molecular results, more frequent identification of actionable targets than tissue testing alone, and accelerated time to treatment for all patients with nonsquamous NSCLC participating in the study. Nearly one-fourth of patients (23%) started treatment before tissue molecular testing was available, similar to the pilot study by Cui et al,^32^ allowing for prompt treatment initiation when needed for symptomatic or deteriorating patients. As many as 70% of patients with advanced nonsquamous NSCLC in the ACCELERATE cohort could have started treatment based on ctDNA results, tissue diagnosis, and PD-L1 tumor assessment. These patients include those with actionable alterations but also those with nonactionable driver alterations, such as *KRAS* non-G12C variants. The detection of these molecular alterations in plasma is informative and can also help guide treatment decisions.

### Limitations

Our study has limitations, including its single-group prospective design and the fact that it was conducted at a single institution. Despite expert selection of patients with radiologic evidence of advanced lung cancer by a multidisciplinary team, only 70% of patients had biopsy-proven advanced NSCLC, while 30% had other diagnoses or no malignant neoplasms. These results reinforce the need for tissue biopsy for lung cancer diagnosis and pathologic subtyping: plasma first does not mean plasma only.^19^ Plasma ctDNA-based NGS has lower sensitivity than tissue-based NGS, with low levels of ctDNA available for analysis, limited sensitivity for detection of fusions, and challenges in assessment of copy number variation. Cell-free RNA assays may yield higher sensitivity.^35^ We believe our results may also have been subject to the Hawthorne effect and affected by the COVID-19 pandemic. Time from referral to tissue biopsy was shorter among the ACCELERATE cohort compared with the reference group (12 days vs 24 days), which suggests a potential effect of the COVID-19 pandemic on access to care when patients with suspected metastatic disease were prioritized for diagnostic tests. ACCELERATE participants were also prioritized for investigation and assessment, including expedited biopsies and rapid medical oncology assessment after ctDNA results. The effect of multidisciplinary diagnostic assessment programs in shortening wait times for diagnosis and treatment of lung cancer, rather than sequential assessment by clinicians, is also highlighted in our study.

**Figure 3.**
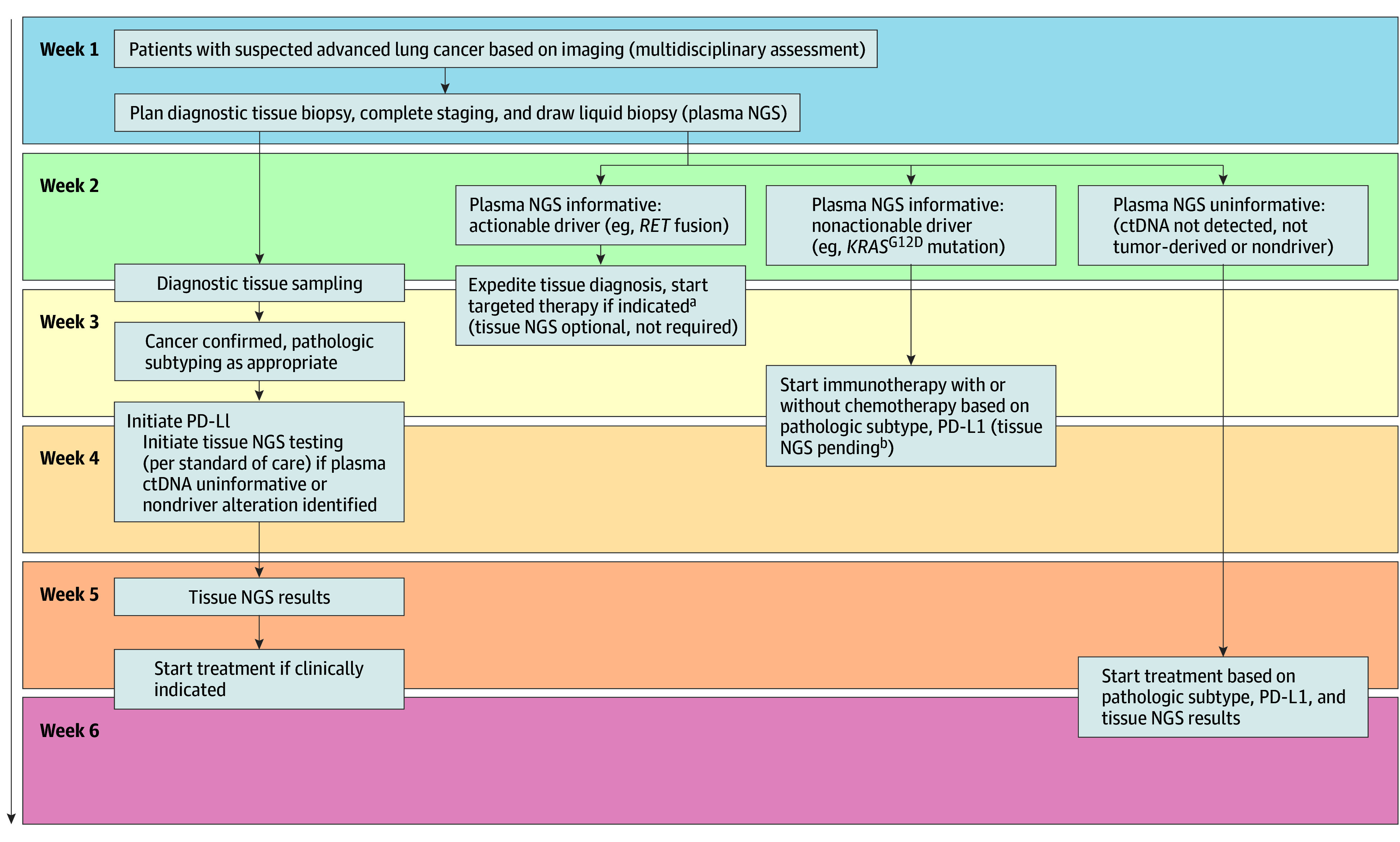
Plasma-First Approach Proposed Algorithm ctDNA indicates circulating tumor DNA; NGS, next-generation sequencing; and PD-L1, programmed cell death ligand 1. ^a^Prioritize tissue sampling (required), pathologic diagnosis, and subtyping (if possible) prior to treatment initiation based on ctDNA results. ^b^Some may consider tissue NGS testing optional in this scenario as the frequency of actionable coalterations is uncommon in the presence of another driver alteration.

Finally, despite the known benefits of liquid biopsy, cost is a potential barrier limiting its implementation. Although 71% of patients in our study had advanced NSCLC, this percentage will vary across geographic regions. Justifying the cost of liquid biopsy for patients without NSCLC may be challenging, although results were potentially informative for some patients with a diagnosis of other cancers. In a cost-effectiveness analysis by Ezeife et al,^36^ the addition of complementary liquid biopsy to tissue testing in patients with newly diagnosed advanced nonsquamous NSCLC did not increase overall treatment costs and led to more patients receiving appropriate targeted therapy. In our study, we provide a possible strategy to optimize molecular testing in NSCLC and time to treatment using plasma ctDNA prior to diagnosis. This approach may permit diagnostic cancer tissue sparing in some scenarios and decrease treatment delays (Figure 3).

## Conclusions

In this nonrandomized clinical trial, the use of liquid biopsy before lung cancer diagnosis in the initial diagnostic workup of patients with suspected advanced NSCLC was associated with shortened time to treatment and molecular results and yielded a higher rate of detection of actionable alterations. Complementing standard tissue testing with plasma testing before diagnosis could increase access to precision medicine and may improve patient outcomes. Although we believe this is a promising strategy to improve the diagnostic journey and treatment decision-making for patients with advanced NSCLC, the effect of this approach on clinically meaningful outcomes, such as quality of life, survival, and cost-effectiveness, still needs to be demonstrated.

## Notes

Supplement 1.
Trial Protocol

Supplement 2. **eFigure 1.** Molecular Profiling Panels **eTable 1.** Tissue Biopsy and Molecular Testing Methods Performed **eFigure 2.** Molecular Alterations in Plasma, Tissue or Both **eTable 2.** Comparison of Tissue Versus ctDNA Results for the Guideline-Recommended Biomarkers in Advanced Non-Squamous NSCLC in the ACCELERATE Cohort (n=90) **eTable 3.** Variants Detected Using ctDNA Testing in Patients Other Cancer Diagnoses in ACCELERATE (n=18)

Supplement 3.
Data Sharing Statement

## References

[1] Hendriks LE, Kerr KM, Menis J, ; ESMO Guidelines Committee. Oncogene-addicted metastatic non–small-cell lung cancer: ESMO Clinical Practice Guideline for diagnosis, treatment and follow-up. Ann Oncol. 2023;34(4):339-357. doi:10.1016/j.annonc.2022.12.009 36872130

[2] National Comprehensive Cancer Network. NCCN Clinical Practice Guidelines in Oncology (NCCN Guidelines): non–small cell lung cancer: version 3.2023. Published 2023. Accessed June 21, 2023. https://www.nccn.org/professionals/physician_gls/pdf/nscl.pdf

[3] Kalemkerian GP, Narula N, Kennedy EB, . Molecular testing guideline for the selection of patients with lung cancer for treatment with targeted tyrosine kinase inhibitors: American Society of Clinical Oncology endorsement of the College of American Pathologists/International Association for the Study of Lung Cancer/Association for Molecular Pathology Clinical Practice Guideline update. J Clin Oncol. 2018;36(9):911-919. doi:10.1200/JCO.2017.76.7293 29401004

[4] Gutierrez ME, Choi K, Lanman RB, . Genomic profiling of advanced non–small cell lung cancer in community settings: gaps and opportunities. Clin Lung Cancer. 2017;18(6):651-659. doi:10.1016/j.cllc.2017.04.004 28479369

[5] Lim C, Tsao MS, Le LW, . Biomarker testing and time to treatment decision in patients with advanced nonsmall-cell lung cancer. Ann Oncol. 2015;26(7):1415-1421. doi:10.1093/annonc/mdv208 25922063

[6] Nadler E, Vasudevan A, Wang Y, Ogale S. Real-world patterns of biomarker testing and targeted therapy in de novo metastatic non–small cell lung cancer patients in the US oncology network. Cancer Treat Res Commun. 2022;31:100522. doi:10.1016/j.ctarc.2022.100522 35189530

[7] Gordan LN, Diaz M, Patel AJ, . Effective biomarker testing rates in a large U.S. community practice. J Clin Oncol. 2022;40(16)(suppl):e21093. doi:10.1200/JCO.2022.40.16_suppl.e21093

[8] Brocken P, van der Heijden EHFM, Oud KTM, . Distress in suspected lung cancer patients following rapid and standard diagnostic programs: a prospective observational study. Psychooncology. 2015;24(4):433-441. doi:10.1002/pon.3660 25201175

[9] Lim C, Sung M, Shepherd FA, . Patients with advanced non-small cell lung cancer: are research biopsies a barrier to participation in clinical trials? J Thorac Oncol. 2016;11(1):79-84. doi:10.1016/j.jtho.2015.09.006 26762742

[10] Kasymjanova G, Small D, Cohen V, . Lung cancer care trajectory at a Canadian centre: an evaluation of how wait times affect clinical outcomes. Curr Oncol. 2017;24(5):302-309. doi:10.3747/co.24.3611 29089797 PMC5659151

[11] Common JL, Mariathas HH, Parsons K, . Reducing wait time for lung cancer diagnosis and treatment: impact of a multidisciplinary, centralized referral program. Can Assoc Radiol J. 2018;69(3):322-327. doi:10.1016/j.carj.2018.02.001 29880435

[12] Maringe C, Spicer J, Morris M, . The impact of the COVID-19 pandemic on cancer deaths due to delays in diagnosis in England, UK: a national, population-based, modelling study. Lancet Oncol. 2020;21(8):1023-1034. doi:10.1016/S1470-2045(20)30388-0 32702310 PMC7417808

[13] García-Pardo M, Makarem M, Li JJN, Kelly D, Leighl NB. Integrating circulating-free DNA (cfDNA) analysis into clinical practice: opportunities and challenges. Br J Cancer. 2022;127(4):592-602. doi:10.1038/s41416-022-01776-9 35347327 PMC9381753

[14] Aggarwal C, Thompson JC, Black TA, . Clinical implications of plasma-based genotyping with the delivery of personalized therapy in metastatic non–small cell lung cancer. JAMA Oncol. 2019;5(2):173-180. doi:10.1001/jamaoncol.2018.4305 30325992 PMC6396811

[15] Leighl NB, Page RD, Raymond VM, . Clinical utility of comprehensive cell-free DNA analysis to identify genomic biomarkers in patients with newly diagnosed metastatic non–small cell lung cancer. Clin Cancer Res. 2019;25(15):4691-4700. doi:10.1158/1078-0432.CCR-19-0624 30988079

[16] Zugazagoitia J, Ramos I, Trigo JM, . Clinical utility of plasma-based digital next-generation sequencing in patients with advance-stage lung adenocarcinomas with insufficient tumor samples for tissue genotyping. Ann Oncol. 2019;30(2):290-296. doi:10.1093/annonc/mdy512 30535340

[17] García-Pardo M, Aparicio I, Martínez Í, . Brief report: clinical outcomes using plasma-based molecular profiling to guide treatment decisions in patients with advanced NSCLC and limited access to broad tissue testing. Clin Lung Cancer. 2023;24(4):366-370. doi:10.1016/j.cllc.2023.02.003 36842853

[18] Rolfo C, Mack P, Scagliotti GV, . Liquid biopsy for advanced NSCLC: a consensus statement from the International Association for the Study of Lung Cancer. J Thorac Oncol. 2021;16(10):1647-1662. doi:10.1016/j.jtho.2021.06.017 34246791

[19] Makarem M, Leighl NB. Molecular testing for lung adenocarcinoma: is it time to adopt a “plasma-first” approach? Cancer. 2020;126(14):3176-3180. doi:10.1002/cncr.32875 32365225

[20] Garcia-Pardo M, Czarnecka K, Law JH, . Plasma-first: accelerating lung cancer diagnosis and molecular profiling through liquid biopsy. Ther Adv Med Oncol. Published online September 20, 2022. doi:10.1177/17588359221126151 36158638 PMC9500258

[21] World Medical Association. World Medical Association Declaration of Helsinki: ethical principles for medical research involving human subjects. JAMA. 2013;310(20):2191-2194. doi:10.1001/jama.2013.281053 24141714

[22] Campbell JD, Lathan C, Sholl L, . Comparison of prevalence and types of mutations in lung cancers among Black and White populations. JAMA Oncol. 2017;3(6):801-809. doi:10.1001/jamaoncol.2016.6108 28114446 PMC5464986

[23] Pritchett MA, Camidge DR, Patel M, . Prospective clinical validation of the InVisionFirst-Lung circulating tumor DNA assay for molecular profiling of patients with advanced nonsquamous non–small-cell lung cancer. JCO Precis Oncol. 2019;3(3):1-15. doi:10.1200/PO.18.00299 32914040 PMC7450945

[24] Perdrizet K, Stockley T, Law JH, . Non–small cell lung cancer (NSCLC) next generation sequencing (NGS) using the Oncomine Comprehensive Assay (OCA) v3: integrating expanded genomic sequencing into the Canadian publicly funded health care model. J Clin Oncol. 2019;37(15)(suppl):2620. doi:10.1200/JCO.2019.37.15_suppl.262031408415

[25] Hwang DM, Albaqer T, Santiago RC, . Prevalence and heterogeneity of PD-L1 expression by 22C3 assay in routine population-based and reflexive clinical testing in lung cancer. J Thorac Oncol. 2021;16(9):1490-1500. doi:10.1016/j.jtho.2021.03.028 33915250

[26] Fiset PO, Labbé C, Young K, . Anaplastic lymphoma kinase 5A4 immunohistochemistry as a diagnostic assay in lung cancer: a Canadian reference testing center’s results in population-based reflex testing. Cancer. 2019;125(22):4043-4051. doi:10.1002/cncr.32422 31390053

[27] Cheung CC, Smith AC, Albadine R, . Canadian ROS proto-oncogene 1 study (CROS) for multi-institutional implementation of *ROS1* testing in non–small cell lung cancer. Lung Cancer. 2021;160:127-135. doi:10.1016/j.lungcan.2021.08.003 34509095

[28] Mateo J, Chakravarty D, Dienstmann R, . A framework to rank genomic alterations as targets for cancer precision medicine: the ESMO Scale for Clinical Actionability of molecular Targets (ESCAT). Ann Oncol. 2018;29(9):1895-1902. doi:10.1093/annonc/mdy263 30137196 PMC6158764

[29] Chakravarty D, Gao J, Phillips SM, . OncoKB: a precision oncology knowledge base. JCO Precis Oncol. 2017;2017(1):1-16. doi:10.1200/PO.17.00011 28890946 PMC5586540

[30] Cui W, Milner-Watts C, O’Sullivan H, . Up-front cell-free DNA next generation sequencing improves target identification in UK first line advanced non–small cell lung cancer (NSCLC) patients. Eur J Cancer. 2022;171:44-54. doi:10.1016/j.ejca.2022.05.012 35704974

[31] Aggarwal C, Marmarelis ME, Hwang WT, . Association of comprehensive molecular genotyping and overall survival in patients with advanced non-squamous non–small cell lung cancer. J Clin Oncol. 2022;40(16)(suppl):9022. doi:10.1200/JCO.2022.40.16_suppl.9022

[32] Cui W, Milner-Watts C, McVeigh TP, . A pilot of blood-first diagnostic cell free DNA (cfDNA) next generation sequencing (NGS) in patients with suspected advanced lung cancer. Lung Cancer. 2022;165:34-42. doi:10.1016/j.lungcan.2022.01.009 35085982

[33] Thompson JC, Aggarwal C, Wong J, . Plasma genotyping at the time of diagnostic tissue biopsy decreases time-to-treatment in patients with advanced NSCLC—results from a prospective pilot study. JTO Clin Res Rep. 2022;3(4):100301. doi:10.1016/j.jtocrr.2022.100301 35392653 PMC8980884

[34] Cheng ML, Milan MSD, Tamen RM, . Plasma cfDNA genotyping in hospitalized patients with suspected metastatic NSCLC. JCO Precis Oncol. 2021;5(5):726-732. doi:10.1200/PO.21.00029 34994618

[35] Choudhury Y, Cher CY, Ho JM, . A cell-free RNA-based next-generation sequencing (NGS) assay for the detection of actionable gene fusions in patients with non–small cell lung cancer (NSCLC). J Clin Oncol. 2022;40(16)(suppl):3040. doi:10.1200/JCO.2022.40.16_suppl.3040

[36] Ezeife DA, Spackman E, Juergens RA, . The economic value of liquid biopsy for genomic profiling in advanced non–small cell lung cancer. Ther Adv Med Oncol. Published online July 26, 2022. doi:10.1177/17588359221112696 35923926 PMC9340413

