# Error in Byline

**DOI:** 10.1001/jamanetworkopen.2025.1147

**Published:** 2025-02-14

In the Original Investigation titled “Association of Circulating Tumor DNA Testing Before Tissue Diagnosis With Time to Treatment Among Patients With Suspected Advanced Lung Cancer: The ACCELERATE Nonrandomized Clinical Trial,”^1^ published July 25, 2023, the 13th author’s degree was incorrect in the byline. Her degree was listed as MD; however, the highest degree that she had earned in 2023 was an honours bachelor of science (HBSc). This article has been corrected.^1^
