# Supplementary material for: Association of Circulating Tumor DNA Testing Before Tissue Diagnosis With Time to Treatment Among Patients With Suspected Advanced Lung Cancer: The ACCELERATE Nonrandomized Clinical Trial
Source: JAMA Netw Open. 2023 Jul 25;6(7):e2325332. doi: 10.1001/jamanetworkopen.2023.25332 (PMC10369925; doi:10.1001/jamanetworkopen.2023.25332)
Supplement: Supplement 1. — Trial Protocol [file jamanetwopen-e2325332-s001.pdf]

CLINICAL STUDY PROTOCOL

TITLE: Accelerating Lung Cancer Diagnosis through Liquid Biopsy (ACCELERATE)

Principal Investigator: Dr. Natasha Leighl
Division of Medical Oncology
Princess Margaret Cancer Centre/University Health Network
7-913 700 University Avenue
Toronto ON M5G 1Z5
T: 416-946-4645; F: 416-946-6546
E: Natasha.Leighl@uhn.ca

Co-Investigators (all UHN):
Dr. Kasia Czarnecka, Division of Thoracic Surgery
Dr. Thomas Waddell, Division of Thoracic Surgery
Dr. Tracy Stockley, Laboratory Medicine and Pathology
Dr. Prodipto Pal, Laboratory Medicine and Pathology
Dr. Patrik Rogalla, Joint Dept. Medical Imaging

Study Fellow: Dr. Miguel Garcia, Division of Medical Oncology

Project Manager: Ms. Jennifer Law, MSc, Division of Medical Oncology
E: Jennifer.Law@uhn.ca

Statistician: Ms. Lisa Le, MSc, Dept. Biostatistics, PM Cancer Centre
E: Lisa.Le@uhnresearch.ca

TABLE OF CONTENTS

LIST OF ABBREVIATIONS/TERMINOLOGY 3
PROTOCOL SUMMARY 4
1. BACKGROUND 6
1.1. NSCLC and Targeted Therapy 6
1.2. Wait Times in NSCLC 6
1.3. Liquid Biopsy and Next-Generation Sequencing 7
1.4. Lung Rapid Assessment and Management Program 8
1.5. Impact of the COVID-19 Pandemic 8
1.6. Rationale for the study 9
2. STUDY OBJECTIVES 9
2.1. Primary Objective 9
2.2. Secondary Objectives 9
2.3. Exploratory Objectives 10
3. METHODS 10
3.1. Liquid Biopsy 10
3.2. Data Collection 11
4. SELECTION OF SUBJECTS 11
4.1. Inclusion Criteria: 11
4.2. Exclusion Criteria: 11
5. STUDY PROCEDURES AND OBSERVATIONS 12
5.1. Schedule of Procedures and Observations 12
5.2. Screening Period 12
5.3. Pre-Study Period (can be same day as screening) 12
5.4. Study Period 12
5.5. Discontinuation of Study and Study Follow-Up 12
5.6. Study Calendar 13
6. STUDY DESIGN, STATISTICAL ANALYSIS, SAMPLE SIZE CALCULATION, AND ACCRUAL PROJECTIONS 13
6.1. Study Design 13
6.2. Statistical Analyses 13
6.3. Sample Size Calculation 14
6.4. Accrual 15
7. ETHICS 15
7.1. Obtaining Informed Consent 15
7.2. Research Ethics Board Review 15
7.3. Data Handling and Record Keeping 15
8. ANTICIPATED RESULTS AND CONCLUSIONS 16
9. STUDY SIGNIFICANCE 16
10. PUBLICATION POLICY 16
11. REFERENCES 17
12. FIGURES 19

LIST OF ABBREVIATIONS/TERMINOLOGY

Abbreviation or special term	Explanation
ALK	Anaplastic Lymphoma Kinase
BRAF	Human gene that encodes a protein called B-Raf
CADTH	Canadian Agency for Drugs and Technologies In Health
CAPCA	Canadian Agency for Provincial Cancer Agencies
CNV	Copy Number Variation
CCO	Cancer Care Ontario
ctDNA	Circulating Tumour Deoxyribonucleic Acid (DNA)
DAP	Diagnostic Assessment Program
EGFR	Epidermal Growth Factor Receptor
EQ5D-5L	Self-assessed, health related, quality of life questionnaire
ERBB2	Erb-B2 Receptor Tyrosine Kinase 2
Indels	Insertions and deletions
KRAS	Human gene that encodes a protein called K-Ras
Lung RAMP	Lung Rapid Assessment and Management Program
MCC	Multidisciplinary Cancer Conference
MET	Mesenchymal Epithelial Transition
NCCN	National Comprehensive Cancer Network
NGS	Next Generation Sequencing
NRG	Neuregulin
NSCLC	Non-Small Cell Lung Carcinoma
PCR	Polymerase Chain Reaction
PD-L1	Programmed Death-Ligand 1
REB	Research Ethics Board
RECIST 1.1	Response Evaluation Criteria in Solid Tumors 1.1
RET	Rearranged During Transfection
ROS-1	ROS proto-oncogene 1
SNV	Single Nucleotide Variant
SOC	Standard of Care
TKI	Tyrosine Kinase Inhibitor
TRK	Tropomyosin Receptor Kinase
UHN	University Health Network
US FDA	United States Food and Drug Administration

74 PROTOCOL SUMMARY

Title of study: Accelerating Lung Cancer Diagnosis through Liquid Biopsy (ACCELERATE)

Sample size: N=175

Study Population: Advanced lung cancer

Study Design: This is a prospective single arm, non-therapeutic, minimally invasive study

Study Duration: This study will begin recruiting in November 2020. Accrual will occur over 12-18 months with study follow-up completing by April 2023.

Main Criteria for Inclusion/Exclusion:

Inclusion Criteria:

Patients referred to the UHN Lung RAMP program with radiologic evidence of advanced disease (incurable stage III or IV) will be eligible, if the following criteria are met:

1. Radiologic (clinical) evidence of advanced, incurable lung cancer;
2. Measurable disease (presumed malignant) by RECIST 1.1;
3. Age≥18 years;
4. Ability to provide written informed consent;
5. Diagnostic biopsy and molecular profiling ordered or planned. Patients remain eligible even if biopsy or tumour testing later fails or is deemed not feasible.

Exclusion Criteria:

1. Pregnancy;
2. Concurrent active malignancy except for localized non-melanomatous skin cancer or non-invasive cervical cancer. Any previous cancer (excluding NSCLC) must have been treated more than 2 years prior to study entry with no current evidence of active disease.

Objectives:

Primary:

1. To compare time to treatment initiation in advanced NSCLC patients for those that had a liquid biopsy compared to patients referred in the previous 12 months that meet the eligibility criteria.

Secondary:

2. To compare time to treatment initiation in a subgroup of patients with advanced non-squamous NSCLC with a smoking history of ≤15 pack years.
3. To evaluate result turnaround time of liquid biopsy compared to standard of care tissue biopsy and testing from time of Lung RAMP referral.
4. To evaluate concordance between liquid and tissue for identification of actionable targets.
5. To model cost-effectiveness of upfront use of liquid biopsy to triage advanced NSCLC to medical oncology compared to current standard of care costs.

Exploratory:

6. To model the impact of using liquid biopsy (“blood-first”) to permit diagnostic cancer tissue sparing.
7. To explore treatment outcomes using liquid biopsy including response and progression-free survival with targeted therapy, and association with time to treatment initiation.

1. BACKGROUND

1.1. *NSCLC and Targeted Therapy*

Lung cancer is the most frequently diagnosed cancer worldwide and the leading cause of cancer-related mortality in both men and women [1]. Non-small cell lung cancer (NSCLC) accounts for 80-85% of all lung cancers and several treatments with differing therapeutic mechanisms are approved for use.

In the current era of targeted therapy, treatment decisions in the first-line advanced NSCLC setting require knowledge of molecular alterations in order to direct cancer therapy [2-4]. The funded standard of care (SOC) for testing in Ontario includes EGFR mutations, ALK translocations and PD-L1 status, to help select patients for targeted or immune therapy [5]. However, there are multiple other genomic markers with associated therapies that are already approved, such as crizotinib or entrectinib for patients with ROS1 rearranged NSCLC, dabrafenib/trametinib for those with BRAF V600E mutations, osimertinib for treatment of the acquired EGFR T790M resistance mutation and larotrectinib for those with TRK fusions.

In addition, the United States Food and Drug Administration (US FDA) has recently approved selpercatinib for RET translocated NSCLC [6] and capmatinib for those with MET exon14 skipping mutations [7]. Thus, at the present time, there are seven oncogenic drivers in lung cancer with an approved targeted therapy by the FDA, and promising novel agents targeting other NSCLC driver mutations, including in ERBB2 or KRAS or NRG fusions, are under investigation [8,9]. The National Comprehensive Cancer Network (NCCN) guidelines for NSCLC recommend genomic assessment for mutations in EGFR, BRAF, MET, ERBB2 and ALK, ROS1, RET and TRK rearrangements, as well as resistance mutations such as EGFR T790M upon progression during EGFR inhibitor therapy [2].

The gold standard for NSCLC diagnosis and molecular testing is tumour tissue genotyping. However, between 15-40% of lung cancer patients do not enough tissue for successful molecular testing [10]. In addition, because of delays obtaining tumour tissue, pathology review and molecular testing results, most patients do not have test results at the time of oncology consultation. This results in prolonged time to treatment for patients, as well as missed treatment opportunities. For example, because of delays in receiving molecular test results, 19% of patients that could have accessed targeted therapy proceed with chemotherapy instead, and another 13% require repeat biopsies, which further delays time to treatment start [11]. Further, many advanced NSCLC patients are not well enough to undergo a repeat biopsy, nor are many well enough to wait for delayed results.

1.2. *Wait Times in NSCLC*

For advanced non-small cell lung cancer (NSCLC) patients, clinical outcomes are directly impacted by time from symptom onset to initiation of appropriate treatment. Unfortunately, for most lung cancer patients, the cancer journey is drawn out with many delays until diagnosis and treatment. The time from the initiation of referral to a cancer centre until treatment can take more than 12 weeks for patients with advanced NSCLC (Figure 1) [12]. This includes waiting for diagnostic tests such as imaging, biopsy, pathology, immunohistochemistry and genomic testing, as well as clinical assessment. Despite rapid diagnostic programs that have been designed to address these issues, limited resources including biopsy bookings, pathology and testing delays remain challenging. Further, these delays are exacerbated by the recent COVID-19 pandemic that has impacted all aspects of healthcare delivery.

Time to diagnosis can vary widely, as this is a complex and multi-dimensional variable. We have learned that prolonged time from symptoms to treatment decision significantly reduces the chance for a patient to access precision medicine, and in some cases, any treatment. The “wait time” to treatment from the patient’s point of view is a period of uncertainty that feeds fear and anxiety [13]. It also has a detrimental impact on patient outcomes, with fewer patients able to access effective therapy and consequently reduced survival.

1.3. Liquid Biopsy and Next-Generation Sequencing

Liquid biopsies are simple, non-invasive blood tests to detect circulating tumour DNA (ctDNA) and have been shown to be non-inferior to tumour tissue genotyping in lung cancer [14]. Next-generation sequencing (NGS) of ctDNA using a hybrid capture approach detects aberrations multiple targetable genes simultaneously, including mutations, fusions and copy number variation. This helps to determine the oncogenes potentially driving the growth of a patient’s malignancy and their potential to benefit from targeted therapy, however liquid biopsy is faster and easier to obtain than standard tissue biopsy [14-17]. Molecular information from liquid biopsies can help oncologists diagnose and discover molecular targets, select targeted treatments, predict response to treatment and monitor for disease recurrence. Prior clinical data have demonstrated the sensitivity of plasma ctDNA testing for the detection of common driver mutations in NSCLC [14-17]. In practice, blood-based profiling requires fewer resources than tissue genotyping and is less invasive. This results in greater patient safety, convenience, and the potential for cost savings. While liquid biopsies at lung cancer diagnosis are not standard of care in all patients, they clearly benefit patients with insufficient tissue for standard molecular analysis or those with undergenotyped samples [18].

Studies have demonstrated that liquid biopsy captures tumour heterogeneity to a greater extent than tumour biopsy. However, challenges with sensitivity of liquid biopsy include the need for tumour measuring 1 cm^3 or more in order to detect ctDNA. Other factors associated with successful detection of ctDNA including the presence of poly-metastatic disease and active disease outside sanctuary compartments like the CNS. In addition, 10-15% of patient tumours do not shed ctDNA into the circulation, known as “non-shedding” tumours. Approximately 10-15% of actionable mutations are identified in liquid biopsy but not in tissue and vice versa. Similar response rates with targeted therapy are seen whether the targetable alteration is found in blood or tissue [14,18-21].

Currently, liquid biopsy is routinely used to detect resistance to EGFR targeted therapy (EGFR T790M resistance mutation). If no mutation is identified in the liquid biopsy, a tumour biopsy is required. Using liquid biopsy as a first step, approximately 40% of patients do not require a subsequent tumour biopsy, with an associated savings in cost, less risk to patients and accelerated time to treatment. At diagnosis of stage IV lung adenocarcinoma, it has been demonstrated that liquid biopsy is non-inferior to tissue testing [14]. It is also faster, and more likely to identify targetable alterations when used as an initial diagnostic approach as versus tumour biopsy as the initial approach (87% versus 67%, p<0.01) [14]. Based on this, the potential for liquid biopsy to accelerate diagnosis and time to treatment in patients with advanced NSCLC presents a major opportunity to improve our current system.

InVisionFirst®-Lung is a validated, rapid, highly sensitive commercial liquid biopsy assay that detects single nucleotide variants (SNV), small insertions and deletions (indels) in mutation hotspots, relevant fusions and copy number variation (CNV) in relevant genomic targets in lung cancer. This 37-gene assay includes key actionable targets in lung cancer, including *EGFR, ALK, ROS1, BRAF, NTRK1, RET, MET, KRAS, ERBB2* and *STK11* among others.

High concordance, 98% with tissue profiling has been reported, with 26% more actionable alterations than standard of care testing [22]. The InVision Platform can successfully detect SNVs, indels, CNVs and fusions in cell free DNA with a variant allelic fraction as low as 0.1% (mean read depth 70,000).

1.4. Lung Rapid Assessment and Management Program

The Lung Rapid Assessment and Management Program (Lung RAMP) is the University Health Network Lung Diagnostic Assessment Program (DAP) supported by Cancer Care Ontario. Lung RAMP aims to quickly and appropriately assess, diagnose and manage patients with presumed lung cancer in the shortest possible timeframe. The Lung RAMP Program has significantly improved the care of lung cancer patients at the University Health Network and in Ontario. What was previously an uncoordinated individual referral process is now a coordinated system capable of providing timely, efficient, coordinated care for patients. The referring physician calls 1-866-LUNG-911 and provides important patient information that avoids delays or repetition of diagnostic tests. The patient is called within 24 hours with a diagnostic and/or treatment plan. The diagnostic workup and referral to the relevant oncology team is expedited, in order to minimize wait times. However, the program has been a victim of its own success, growing from ~350 referrals per year to over 900 per year, with few or no additional resources allocated (Figure 2A). Most of these patients (~2/3), are referred with advanced disease and are not surgical candidates (Figure 2B), but still require pathologic diagnosis and staging. Prior to the COVID-19 pandemic, wait times for imaging were less than 28 days, while wait times for biopsy ranged from 28 to 32 days (Figure 3). These have both lengthened significantly during the pandemic. In addition, the time from biopsy to molecular profiling results was a mean of 28 days pre-COVID but this has also lengthened. Thus, patients can wait approximately 3 months, or potentially longer during the pandemic and recovery, in order to have complete lung cancer staging, a pathologic diagnosis and all molecular results available in order to start treatment. Given the current dearth of resources allocated for biopsy slots and pathologists, the system requires an innovative approach to shorten current wait times, accelerate time to treatment and promote access to personalized medicine for more of Ontario’s patients with lung cancer.

1.5. Impact of the COVID-19 Pandemic

Clinical activity throughout Canadian hospitals, including at the University Health Network, has been markedly restricted to protect patients and providers during the COVID-19 pandemic. This has served to further exacerbate the pre-existing challenges with timely diagnosis and access to cancer treatment for lung cancer patients, including at UHN. In addition, with shortages of personal protective equipment and longer space ventilation requirements, fewer procedures can be performed on a daily basis. This has led to significant delay in time to biopsy, with subsequent delays in time to diagnosis and molecular profiling results. Currently, diagnostic volumes for lung cancer appear to have been reduced by at least 25% [personal communication, Cancer Care Ontario Data Request]. The present study will accelerate patient access to precision medicine, through allowing rapid molecular diagnosis with plasma ctDNA testing in the face of growing wait times for routine imaging, tumour biopsy, pathologic diagnosis and molecular testing. The potential for a “blood-first” approach to decrease the number of invasive lung biopsies will not only decrease risks for patients, but also for health care providers. The specialist teams that perform these invasive

procedures are at high risk for COVID-19 transmission during bronchoscopy and/or interventional biopsy, including transthoracic sampling (image-guided biopsies).

1.6. Rationale for the study

The purpose of this trial is to prospectively assess the utility of blood-based next generation sequencing to accelerate time to treatment for newly diagnosed patients with advanced NSCLC, compared to conventional molecular tumour testing.

Many other studies [14,17, 21-25] have already demonstrated that plasma-based NGS genotyping is feasible, rapid and useful in the clinical practice setting for patients with advanced NSCLC.

Plasma NGS used in patients with newly diagnosed advanced NSCLC successfully identifies guideline recommended biomarkers at a rate at least as high as SOC tissue testing and returns these results significantly faster and for a significantly higher proportion of the population. Moreover, ctDNA-detected guideline recommended biomarkers were invariably present in tissue, when tissue was successfully tested, reinforcing that ctDNA genotyping results may be used in clinical management in the same way tissue genotyping results are currently used [9, 14]

These results suggest that initial biomarker assessment using ctDNA rather than tissue ("blood-first"), reserving tissue for PD-L1 IHC and reflex testing when ctDNA is negative for any known oncogenic driver mutations, will improve the biomarker discovery rate, turn-around time and decrease time to treatment. The net result will be to increase the number of patients with newly diagnosed advanced NSCLC that will receive guideline complete biomarker testing and be able to access a precision medicine approach to their cancer therapy [14]. The acceleration of time to diagnosis and treatment is expected to favorably impact outcomes in this population.

Based on these promising results, in this study we will analyze if the "blood-first" approach can accelerate time to treatment compared with the standard diagnostic pathway including tissue genotyping.

2. STUDY OBJECTIVES

This study will assess the utility of liquid biopsy to accelerate time to treatment for selected patients with radiographic evidence of advanced lung cancer (Figure 1).

2.1. Primary Objective

1. To compare time to treatment initiation in advanced NSCLC patients for those that had a liquid biopsy compared to patients referred in the previous 12 months that meet the eligibility criteria.

   Hypothesis: Wait times to treatment initiation will be reduced up to 50% (4-6 weeks) for patients who have a liquid biopsy with actionable lung cancer targets.

2.2. Secondary Objectives

1. To compare time to treatment initiation in a subgroup of patients with advanced non-squamous NSCLC with a smoking history of ≤15 pack years.

2. To evaluate result turnaround time of liquid biopsy compared to standard of care tissue biopsy and testing from time of Lung RAMP referral.

   Hypothesis: Molecular results from liquid biopsy will be faster than standard of care tissue profiling.

3. To evaluate concordance between liquid and tissue for identification of actionable targets.

   Hypothesis: We hypothesize that liquid and tissue molecular results will be comparable assuming use of broad based NGS panel testing for both.

4. To model cost-effectiveness of upfront use of liquid biopsy to triage advanced NSCLC to medical oncology compared to current standard of care costs.

   Hypothesis: Upfront liquid biopsy will save biopsy costs in a subset of patients, compared to similar profiling in tumour tissue.

2.3. Exploratory Objectives

1. To model the impact of using liquid biopsy ("blood-first") to permit diagnostic cancer tissue sparing.
2. To explore treatment outcomes using liquid biopsy including response and progression-free survival with targeted therapy.

3. METHODS

This is a prospective single arm, non-therapeutic, minimally invasive study which will conducted at the University Health Network (UHN). One hundred and fifty patients will be accrued over an estimated 12-18 months. Based on current Lung RAMP referral patterns and demographics, one third will be never smokers (target subgroup accrual N=40).

Eligible patients will be identified through the weekly Lung RAMP MCC and contacted by the study coordinator. Consenting patients would undergo liquid biopsy (plasma ctDNA testing) in addition to standard of care imaging, tumour biopsy and tumour tissue molecular profiling through Lung RAMP. Patients with non-diagnostic tumour biopsies or insufficient tumour tissue for molecular profiling would also be eligible to participate.

Consenting patients would undergo liquid biopsy during their first or next onsite visit to UHN (e.g. imaging, standard of care blood tests or medical assessment). Whenever possible, the liquid biopsy would be added to a planned blood draw for standard of care, avoiding additional venipuncture for the patient. A total of 4 Streck tubes of blood (40 mL total) will be drawn. Samples will be de-identified and labelled with a study code.

3.1. Liquid Biopsy

Consenting patients will undergo peripheral blood draw (approximately 40 mL) collected in Streck™ tubes or kits provided by Inivata. Two tubes (20 mL) will be shipped to Inivata (North Carolina, USA) for InVisionFirst® Lung profiling in real time. The remaining 2 tubes will be sent to the Advanced Molecular Diagnostics Laboratory (AMDL) at Princess Margaret Cancer Centre for nucleic acid extraction and mutation profiling using the Oncomine™ Pan-Cancer

Cell-Free Assay (ThermoFisher). This panel can detect lung tumour-derived clinically-relevant SNVs, small indels and fusions.

3.2. Data Collection

Molecular profiling results from both tumour and ctDNA will be recorded, and incremental actionable genomic targets will be identified for each method. Any complications from either the liquid or tumour biopsy will be captured, as well as any failure of tissue or blood sampling, molecular profiling and repeat biopsies.

Turnaround time for molecular results for liquid and tumour tissue biopsy, time to treatment initiation, treatment received and outcomes including tumour response, progression-free and overall survival will be collected.

Patients will be asked to complete the EQ-5D-5L at baseline and at 3 months to assess quality of life. Patients may complete the EQ-5D-5L survey by phone or by mail if they do not have a routine (in person) doctor's visit scheduled. Patients that are unable to complete the EQ-5D-5L (literacy, physical issues, unavailable translation) are still eligible to participate.

Patient visits and procedures during the diagnostic work up will be collected. Costs of both diagnostic strategies will be captured using time in motion studies, direct costs from UHN and list costs as appropriate. These will be presented as a cost-consequence analysis of liquid biopsy vs. standard of care tissue biopsy and molecular profiling.

4. SELECTION OF SUBJECTS

Patients referred to the Lung RAMP program will be eligible if they are deemed to have advanced lung carcinoma by imaging. These patients (all cases) are currently discussed at the weekly Lung RAMP Multidisciplinary Cancer Conference (MCC) that includes thoracic surgery, interventional respirology, radiology, radiation and medical oncology representatives. Patients reviewed at MCC who meet the inclusion criteria will be approached to participate in the study as part of their ongoing diagnostic work up.

4.1. Inclusion Criteria:

Patients referred to the Lung RAMP program with radiologic evidence of advanced disease (incurable stage III or IV) will be eligible, if the following criteria are met:

1. MCC or study team confirms radiologic (clinical) evidence of advanced, incurable lung cancer;
2. Measurable disease (presumed malignant) by RECIST 1.1;
3. Age ≥18 years;
4. Ability to provide written informed consent;
5. Diagnostic biopsy and molecular profiling ordered or planned. Patients remain eligible even if biopsy or tumour testing later fails or is deemed not feasible.

4.2. Exclusion Criteria:

1. Pregnancy;
2. Concurrent active malignancy except for localized non-melanomatous skin cancer or non-invasive cervical cancer. Any previous cancer (excluding NSCLC) must

have been treated more than 2 years prior to study entry with no current evidence of active disease.

5. STUDY PROCEDURES AND OBSERVATIONS

5.1. Schedule of Procedures and Observations

All participants must provide written, signed, informed consent using the latest approved version of the Institutional Research Ethics Board informed consent form (ICF). A copy of the signed ICF will be given to the subject. The original will be kept on file in study records. Study overview is shown in Figure 4.

5.2. Screening Period

The following procedures and assessments must be completed prior to pre-study assessment:

- MCC review and confirmation of eligibility
- Informed Consent

5.3. Pre-Study Period (can be same day as screening)

- Blood collection into 4 Streck cell preservation tubes (40 mL)
- Baseline quality of life assessment using the EQ-5D-5L

5.4. Study Period

Patients will proceed with standard of care diagnostic work up, imaging, tumour biopsy and tumour tissue molecular profiling through Lung RAMP. There may be some variability in timing of scans and tests.

- Repeat quality of life assessment will be administered at 3 months (12 weeks +/- 4 weeks) from study blood collection using the EQ-5D-5L
- Molecular profiling results from both tumour and ctDNA will be recorded
- Patients will be treated as per standard of care based on tissue and/or blood genotyping results
- Time to treatment initiation and patient visits/procedures will be recorded

5.5. Discontinuation of Study and Study Follow-Up

Patients will be followed for a minimum of 12 months and up to 2 years. Patients will be discontinued from the study when the first of any of the following events occurs:

- At time of death; or
- If the patient withdraws consent; or
- 2 year of follow up; or
- Study closure.

5.6. Study Calendar

Study Visit Day	Screening	Pre-Study Period	Study Period	12 +/- 4 weeks	End of Study[2]
Informed consent<br>Eligibility confirmation<br>Patient Demographics	X				
Quality of Life (EQ-5D-5L)		X[3]		X	
Blood draw		X			
Molecular results<br>Time to treatment initiation			X		
Treatment received			X		X
Treatment outcome (confirmed or unconfirmed response rate by RECIST 1.1, progression-free survival, 1 and 2 year survival)					

[1] Baseline testing, blood draw and quality of life assessment, may be completed on same day after screening procedures

[2] End of study visit should occur not later than study closure or patient consent for follow-up is withdrawn, whichever comes first.

[3]Baseline quality of life assessment should be completed prior to the blood draw when possible.

6. STUDY DESIGN, STATISTICAL ANALYSIS, SAMPLE SIZE CALCULATION, AND ACCRUAL PROJECTIONS

6.1. Study Design

This is a single arm, minimally invasive non-therapeutic study conducted at a single centre.

The time to treatment decision (T_{LB}) in the study cohort by liquid (T_L) and tissue biopsy (T_B) is measured from the date of referral to the earliest date of receiving a liquid or tissue biopsy report indicating actionable genomic aberrations, or $T_{LB} = \min(T_L, T_B)$. The time to treatment decision using tissue biopsy alone (T_B) will be collected in a chart-review comparison cohort (patients referred in the previous 12 months that meet the eligibility criteria). The time to treatment decision by liquid biopsy (T_{LB}) vs by tissue biopsy alone (T_B) will be compared.

6.2. Statistical Analyses

The primary objective is to compare time to treatment initiation in advanced NSCLC patients for those that had a liquid biopsy compared to patients referred in the previous 12 months that meet the eligibility criteria. Time from referral to systemic treatment decision, by tissue biopsy alone vs by liquid and tissue biopsy, will be compared using a two-sample t-test or a Wilcoxon Mann-Whitney test if the normality assumption cannot be satisfied.

The secondary objectives are:

- To compare time to treatment initiation in a subgroup of patients with advanced non-squamous NSCLC with a smoking history of ≤15 pack years;
- To evaluate result turnaround time of liquid biopsy compared to standard of care tissue biopsy and testing from time of Lung RAMP referral within the study cohort using a paired t-test or Wilcoxon signed rank test;
- To evaluate concordance between liquid and tissue for identification of actionable targets within the study cohort; and,
- To model cost-effectiveness of upfront use of liquid biopsy to triage advanced NSCLC to medical oncology compared to current standard of care costs.

Descriptive analysis will be used to describe treatments received and disease outcomes (confirmed or unconfirmed response by RECIST 1.1), including subgroup analyses of actionable genomic alterations (such as *EGFR*). The Kaplan-Meier method will be used to describe progression-free, time to treatment failure and overall survival in the study cohort. Patients will be followed for a minimum of 12 months and up to 24 months.

Change scores in health-related qualify of life and patient utility (EQ5D-5L) between baseline and 3 months will be calculated and summarized for those with actionable genomic aberrations identified in liquid biopsy and those requiring standard of care tissue diagnosis and profiling.

A cost-effectiveness model will be developed comparing the initial use of liquid biopsy versus the current standard of tissue biopsy and profiling from the perspective of the Canadian healthcare system for the horizon of the study period. Study data will be used as model inputs, as well as costs (current CAD) from UHN and published list prices. Additional inputs will be derived from published literature and expert opinion as required. Sub-analysis of resource utilization with each approach will be performed to estimate COVID exposure risk to patients and healthcare providers. In addition, the potential of liquid biopsy to spare tumour tissue will also be derived.

6.3. Sample Size Calculation

It is estimated that ~175 patients/year with advanced lung cancer are referred to the UHN Lung RAMP program for diagnostic work up. Based on current Lung RAMP data, at least 90% of these will have NSCLC subtype, (<10% small cell carcinoma or non-lung cancer pathology), and an additional 5% will decline participation or be ineligible. Thus 150 eligible patients will be seen per year, and the majority will have non-squamous subtype (~85%). Current data indicate that 34% are lifetime never smokers, and molecular testing data indicate that 28.2% of newly diagnosed advanced non-squamous lung cancer patients at UHN have actionable *EGFR, BRAF(V600E)*, *ALK* or *ROS1* aberrations.

The sample size of 175 is a convenience sample to address accrual timelines and risk of non-eligible histologic subtypes (i.e. SCLC, carcinoid, non-lung primary). The subgroup analysis sample of 40 patients with ≤15 pack year smoking history is expected to yield N1=24 patients (59%) with targetable aberrations based on our previous experience in this population of light/never smokers referred to UHN [18]. We expect there are 25-50 patients (min N2=25) in the comparison group referred in the prior 12 months. Assuming a 4-6 week reduction in time to treatment, an equal group size (N1=N2), a standard deviation of 4 for the liquid biopsy group and an unequal standard deviation of 5 for the comparison group to account for variability in getting successful tumour molecular testing, we will achieve a power >80%, at

85%, 96% and 99% respectively for a difference of 4, 5 and 6 weeks, using a two-sided two-sample unequal-variance t-test with significance level (alpha) of 0.05 (Figure 5).

6.4. Accrual

We estimate that 4 eligible patients will be seen in Lung RAMP weekly. Up to 10% of patients may be ineligible, and another 10% may decline. With a projected accrual of 3 patients per week, target accrual is expected by 12-18 months.

7. ETHICS

7.1. Obtaining Informed Consent

It is mandatory that consent be appropriately obtained for each participant/potential participant in accordance with ICH-GCP section 4.8.

Additionally, in accordance with GCP 4.8.2, participants/potential participants may need to be informed of any new information that may impact a participant's/potential participant's willingness to participate in the study.

Based upon applicable guidelines and regulations (Declaration of Helsinki, ICH-GCP), a participating investigator (as defined on the participants list) is ultimately responsible, in terms of liability and compliance, for ensuring informed consent has been appropriately obtained. In accordance with GCP 4.8.5, it is acceptable for the Qualified Investigator to delegate the responsibility for conducting the consent discussion.

Each participant must sign a consent form prior to their enrollment in the study to document his/her willingness to take part. If participants/potential participants are to be informed of new information if it becomes available during the course of the study, communication of this information should be documented.

Translators are permitted to obtain informed consent. If quality of life or other questionnaires are not available in the participant's native language, these may be omitted.

In accordance with ICH-GCP 4.8.9, if a subject is unable to read then informed consent may be obtained by having the consent form read and explained to the subject.

7.2. Research Ethics Board Review

This study protocol, including the informed consent document, will be reviewed and approved by the University Health Network Research Ethics Board before any study related procedures commenced. Review and approval of any amendment to the study will be completed by the REB before any changes are implemented, except those necessary to eliminate an immediate hazard to the study participants.

7.3. Data Handling and Record Keeping

All essential documents must be maintained as per C.05.012 and in accordance with ICH-GCP.

The Qualified Investigator must ensure compliance with the Regulations and the GCP Guideline from every person involved in the conduct of the study at the site.

Essential documents must be retained for 10 years following the completion of the trial (10 years post final analysis, last data collected, or closure notification to REB, whichever is later).

In accordance with GCP 4.9.7, upon request by the monitor, auditor, REB or regulatory authority, the investigator/institution must make all required trial-related records available for direct access.

8. ANTICIPATED RESULTS AND CONCLUSIONS

We anticipate that molecular results from liquid biopsy will identify actionable mutations for patients with advanced NSCLC, and that the time to diagnosis and treatment will be accelerated in this population of patients with targetable mutations. This study will demonstrate the potential of liquid biopsy to complement tumour biopsy and facilitate diagnosis and earlier treatment for patients with advanced lung cancer in Ontario.

9. STUDY SIGNIFICANCE

Using liquid biopsy upfront could significantly decrease wait times for molecular diagnosis and give advanced lung cancer patients a faster route to the treatment they need. This could have a meaningful impact on survival rates and quality of life. In addition, patients may become eligible for clinical trials not otherwise available without this testing.

This study will establish liquid biopsy as an important diagnostic complement to tumour biopsy and molecular profiling in patients with advanced NSCLC in the province of Ontario. Not only will it accelerate time to treatment, it will increase the proportion of Ontario's lung cancer patients that are eligible for a precision medicine approach to therapy. These results will apply not only to Ontario's lung cancer patients but will be generalizable to all Canadian lung cancer patients and those in international jurisdictions.

10. PUBLICATION POLICY

The results of this study will be published. The principal and co-invetigators listed in the protocol will be authors. Additional individuals, including from Inivata, up to the maximum permitted by the publication journal, will be those who have made the most significant contribution to the overall success of the study. Final author order will be confirmed by the principal investigator. It will be the responsibility of the principal investigator to ensure write up of the results of the study within six months of its completion. Supporting groups and agencies will be acknowledged.

11. REFERENCES

1. World Health Organization and International Agency for Research on Cancer: GLOBOCAN 2012: estimated cancer incidence, mortality and prevalence worldwide in 2012. Journal aspx, accessed on Jan 27 2015
2. Ellis PM, Vandermeer R. Delays in the diagnosis of lung cancer. J Thorac Dis. 2011 Sep;3(3):183-8.
3. Ettinger DS, Wood DE, Aisner DL, Akerley W, Jessica Bauman, Bharat, Ankit, et al. Non-Small Cell Lung Cancer, Version 3.2020, NCCN Clin Pract Guidel Oncol NCCN Guidel [Internet]. 2018; Available from: https://www.nccn.org/professionals/physician_gls/pdf/nscl.pdf
4. Planchard D, Popat S, Kerr K, et al. Metastatic non-small cell lung cancer: ESMO Clinical Practice Guidelines for diagnosis, treatment and follow-up. Ann Oncol. 2018;29(suppl 4):iv192–iv237.
5. Leighl NB, Rekhtman N, Biermann WA, Huang J, Mino-Kenudson M, Ramalingam SS, West H, Whitlock S, Somerfield MR. Molecular testing for selection of patients with lung cancer for epidermal growth factor receptor and anaplastic lymphoma kinase tyrosine kinase inhibitors: American Society of Clinical Oncology endorsement of the College of American Pathologists/International Association for the Study of Lung Cancer/Association for Molecular Pathology Guideline. J Clin Oncol 2014; 32:3673-9.
6. Cancer Care Ontario Systemic Therapy for NSCLC Guidelines. https://www.cancercareontario.ca/guidelines-advice.
7. Drilon A. et al. PL02.08 Registrational Results of LIBRETTO-001: A Phase 1/2 Trial of LOXO-292 in Patients with RET Fusion-Positive Lung Cancers. Journal of Thoracic Oncology, Volume 14, Issue 10, S6 - S7
8. Wolf J, Takashi S, Ji-Youn H, et al. Capmatinib (INC280) in METΔex14-mutated advanced non-small cell lung cancer (NSCLC): Efficacy data from the phase II GEOMETRY mono-1 study. Journal of Clinical Oncology 37, n.º 15_suppl (20 de mayo de 2019): 9004-9004.
9. Rolfo C, Russo A. HER2 Mutations in Non–Small Cell Lung Cancer: A Herculean Effort to Hit the Target. Cancer Discovery 10, n.º 5, 643.
10. Yang H, Liang SQ, Schmid RA, Peng RW. New Horizons in KRAS-Mutant Lung Cancer: Dawn After Darkness. Frontiers in Oncology 9 (2019): 953. https://doi.org/10.3389/fonc.2019.00953.
11. Lim C, Sekhon HS, Cutz JC, Hwang DM, Kamel-Reid S, Carter RF, Santos GDC, Waddell T, Binnie M, Patel M, Paul N, Chung T, Brade A, El-Maraghi R, Sit C, Tsao MS, Leighl NB. Improving molecular testing and personalized medicine in non-small cell lung cancer in Ontario.
12. Ellis PM, Verma S, Sehdev S, Younus J, Leighl NB. Challenges to implementation of an epidermal growth factor receptor testing strategy for non-small-cell lung cancer in a publicly-funded health care system. J Thorac Oncol 2013; 8:1136-41.
13. Labbé C, Anderson M, Simard S et al. Wait times for diagnosis and treatment of lung cancer: A single-centre experience. Curr Oncol Tor Ont 2017; 24: 367–73.
14. Leighl NB, Page RD, Raymond VM, Daniel DB, Divers SG, Reckamp KL, Villalona-Calero MA, Dix D, Odegaard JI, Lanman RB, Papadimitrakopoulou VA. Clinical Utility of Comprehensive Cell-free DNA Analysis to Identify Genomic Biomarkers in Patients with Newly Diagnosed Metastatic Non-mall Cell Lung Cancer. Clin Cancer Res. 2019 Aug 1;25(15):4691-4700.

15. Weber B, Meldgaard P, Hager H, Wu L, Wei W, Tsai J, Khalil A, Nexo E, Sorensen BS. Detection of EGFR mutations in plasma and biopsies from non-small cell lung cancer patients by allele-specific PCR assays. BMC Cancer 2014. 14, 294.
16. Wan JC, Massie C, Garcia-Corbacho J, Mouliere F, Brenton JD, Caldas C, Pacey S, Baird R, Rosenfeld N. Liquid biopsies come of age: towards implementation of circulating tumour DNA. Nat Rev Cancer. 2017 Apr;17(4):223-238. Epub 2017 Feb 24.
17. Sabari JK, Santini F, Bergagnini I, Lai WV, Arbour KC, Drilon A. Changing the Therapeutic Landscape in Non-small Cell Lung Cancers: the Evolution of Comprehensive Molecular Profiling Improves Access to Therapy. Curr Oncol Rep. 2017 Apr;19(4):24.
18. Makarem M, Leighl NB. Molecular Testing for Lung Adenocarcinoma: Is it Time to Adopt a “Plasma-First” Approach? Cancer 2020; 126(14):3176-80.
19. Meador CB, Oxnard GR. Effective Cancer Genotyping-Many Means to One End. Clin Cancer Res. 2019 Aug 1;25(15):4583-4585.
20. Leighl NB, Kamel-Reid S, Cheema PK, et al. Multicenter Validation Study to Implement Plasma Epidermal Growth Factor Receptor T790M Testing in Clinical Laboratories. JCO Precis Oncol 2020;4;520-533.
21. Mack PC, Banks KC, Espenschied CR, Burich RA, et al. Spectrum of driver mutations and clinical impact of circulating tumor DNA analysis in non–small cell lung cancer: Analysis of over 8000 cases. Cancer. doi:10.1002/cncr.32876
22. Pritchett MA, Camidge DR, Patel M et al. Prospective Clinical Validation of the InVisionFirst-Lung Circulating Tumor DNA Assay for Molecular Profiling of Patients with Advanced Nonsquamous Non-small-cell Lung Cancer. JCO Precis Oncol 2019;3:PO.18.00299.
23. Remon J, Lacroix L, Jovelet C et al. Real-World Utility of an Amplicon-Based Next-Generation Sequencing Liquid Biopsy for Broad Molecular Profiling in Patients With Advanced Non–Small-Cell Lung Cancer. JCO Precision Oncology. 1-14. 10.1200/PO.18.00211.
24. Aggarwal C, Thompson JC, Black TA, Katz SI, Fan R, Yee SS, et al. Clinical Implications of Plasma-Based Genotyping With the Delivery of Personalized Therapy in Metastatic Non-Small Cell Lung Cancer. JAMA Oncol.2018;
25. Li, B. T. et al. Ultra-deep next-generation sequencing of plasma cell-free DNA in patients with advanced lung cancers: results from the actionable Genome Consortium. Ann. Oncol. 30, 597–603 (2019).

12. FIGURES

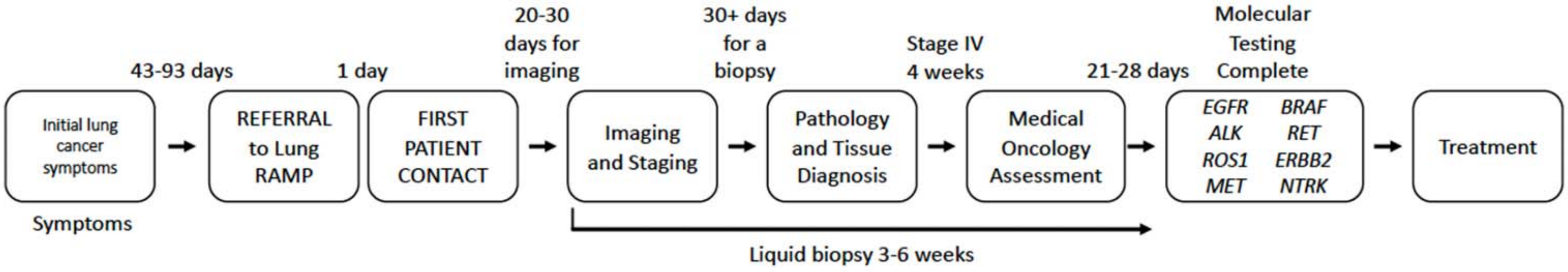

Figure 1: Lung cancer patient journey at Princess Margaret Cancer Centre with current approximate wait times. Proposed study evaluating liquid biopsy at imaging diagnosis for patients with radiologic evidence of Stage IV disease.

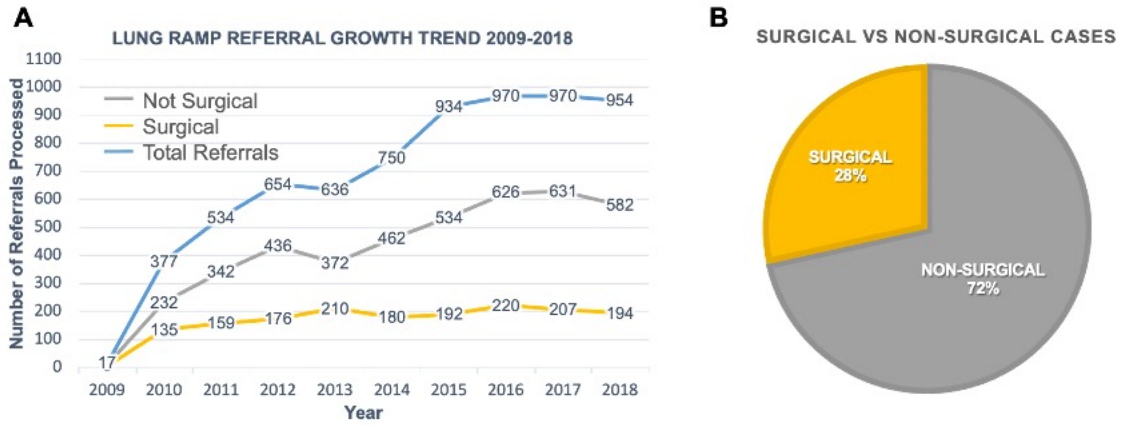

Figure 2: A. Referral growth to the UHN Lung Cancer Rapid Assessment & Management Program (Lung RAMP) from 2009-2018. **B.** Proportion of lung cancer cases referred to Lung RAMP 2009-2018 that were surgical versus non-surgical. [CONFIDENTIAL. Source: UHN Lung-RAMP]

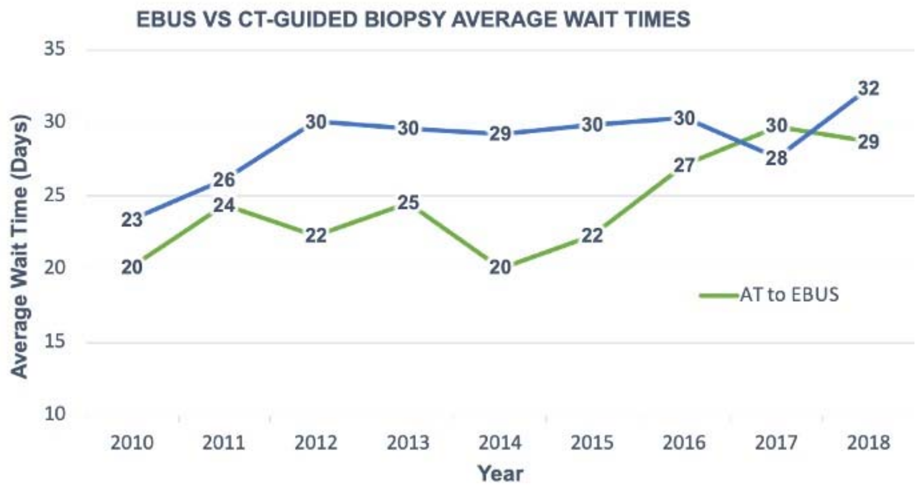

Figure 3: Average wait times (AT) for EBUS versus CT-guided biopsies from 2010-2018. [CONFIDENTIAL. Source: UHN Lung-RAMP]

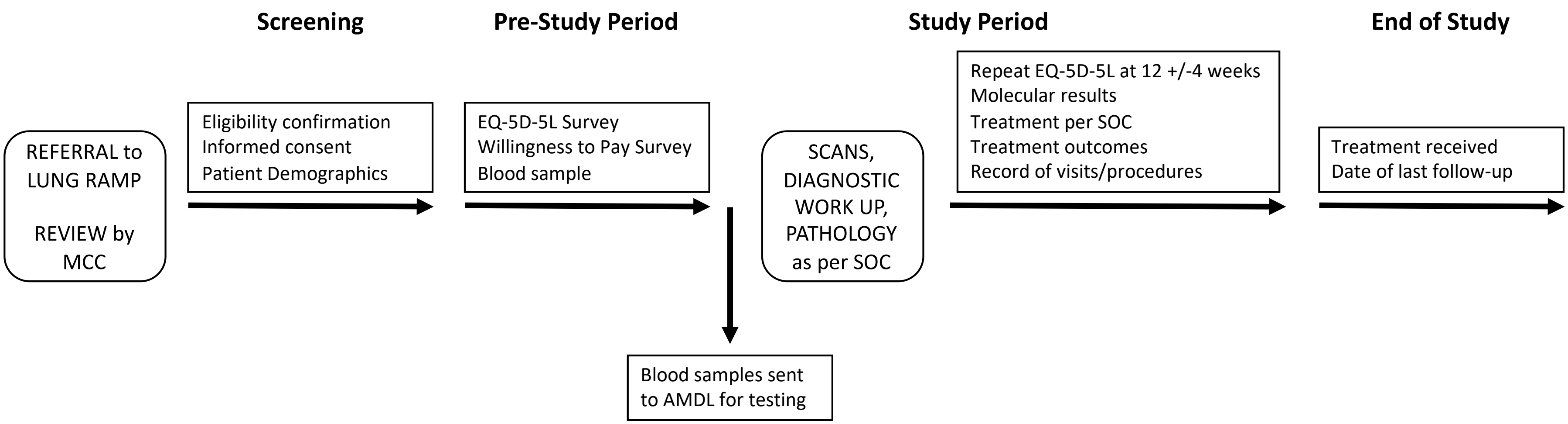

Figure 4: Study schema.

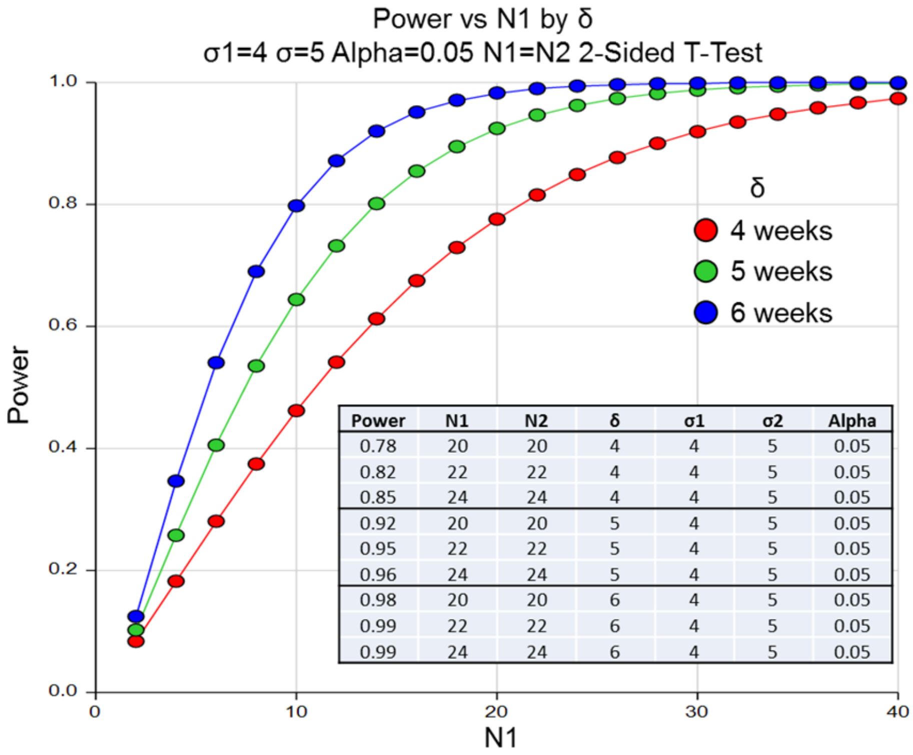

Power	N1	N2	δ	σ1	σ2	Alpha
0.78	20	20	4	4	5	0.05
0.82	22	22	4	4	5	0.05
0.85	24	24	4	4	5	0.05
0.92	20	20	5	4	5	0.05
0.95	22	22	5	4	5	0.05
0.96	24	24	5	4	5	0.05
0.98	20	20	6	4	5	0.05
0.99	22	22	6	4	5	0.05
0.99	24	24	6	4	5	0.05

Figure 5: The subgroup analysis sample of 40 patients is expected to yield 24 patients (N1) with actionable alterations. Comparing to a group from the previous 12 months (N2) and assuming a reduction in 4-6 weeks (δ), we will achieve a power of >80% with this sample size. N1: Number of patients with actionable lung cancer targets in study (group 1). N2: Number of patients with actionable lung cancer targets referred in the preceding 12 months (group 2). σ1: Standard Deviation of group 1; σ2: Standard Deviation of group 2. δ: Difference in time from initiation of referral to treatment (in weeks) between group 1 and group 2.
