# Supplementary material for: Association of Circulating Tumor DNA Testing Before Tissue Diagnosis With Time to Treatment Among Patients With Suspected Advanced Lung Cancer: The ACCELERATE Nonrandomized Clinical Trial
Source: JAMA Netw Open. 2023 Jul 25;6(7):e2325332. doi: 10.1001/jamanetworkopen.2023.25332 (PMC10369925; doi:10.1001/jamanetworkopen.2023.25332)
Supplement: Supplement 2. — eFigure 1. Molecular Profiling Panels eTable 1. Tissue Biopsy and Molecular Testing Methods Performed eFigure 2. Molecular Alterations in Plasma, Tissue or Both eTable 2. Comparison of Tissue Versus ctDNA Results for the Guideline-Recommended Biomarkers in Advanced Non-Squamous NSCLC in the ACCELERATE Cohort (n=90) eTable 3. Variants Detected Using ctDNA Testing in Patients Other Cancer Diagnoses in ACCELERATE (n=18) [file jamanetwopen-e2325332-s002.pdf]

Supplementary Online Content

García-Pardo M, Czarnecka-Kujawa K, Law JH, et al. Association of circulating tumor DNA testing before tissue diagnosis with time to treatment among patients with suspected advanced lung cancer: the ACCELERATE nonrandomized clinical trial. *JAMA Netw Open.* 2023;6(7):e2325332. doi:10.1001/jamanetworkopen.2023.25332

eFigure 1. Molecular Profiling Panels
eTable 1. Tissue Biopsy and Molecular Testing Methods Performed
eFigure 2. Molecular Alterations in Plasma, Tissue or Both
eTable 2. Comparison of Tissue Versus ctDNA Results for the Guideline-Recommended Biomarkers in Advanced Non-Squamous NSCLC in the ACCELERATE Cohort (n=90)
eTable 3. Variants Detected Using ctDNA Testing in Patients Other Cancer Diagnoses in ACCELERATE (n=18)

This supplementary material has been provided by the authors to give readers additional information about their work.

© 2023 García-Pardo M et al. *JAMA Network Open.*

eFigure 1. Molecular Profiling Panels. 1A. InVisionFirst-Lung liquid biopsy tumor profiling panel, InvCore v1.5[22] 1B. Standard-of-care tissue NGS panel in ACCELERATE (Oncomine Comprehensive Assay v3).[23] 1C. Standard-of-care tissue NGS panel in reference cohort (Trusight Tumor 15 Panel, Illumina™).

1A

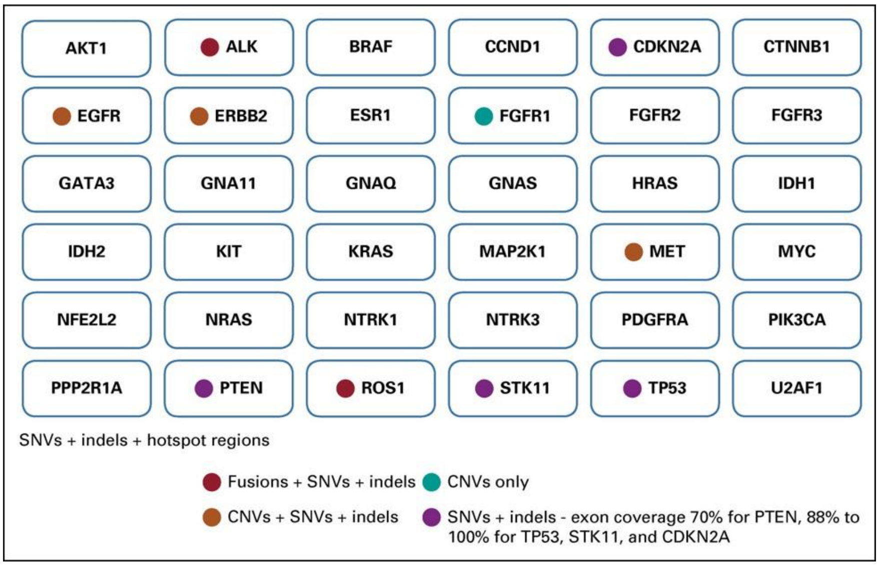

Sensitivity 73.9% specificity 99.8%, positive predictive value (PPV) 97.8% for 8 key genes (*ALK, ROS1* fusions, *BRAF* V600E, *EGFR* exons 18-21, *ERBB2* insertions, *MET* exon 14 splice, *KRAS, STK11* variants).

1B

Hotspot genes				Full-length genes			Copy number genes		Gene fusions (inter- and intragenic)		
AKT1	*ESR1*	*KIT*	*PDGFRB*	*ARID1A*	*FBXW7*	*PTEN*	*AKT1*	*FGFR4*	*AKT2*	*FGFR2*	*NUTM1*
AKT2	*EZH2*	*KNSTRN*	*PIK3CB*	*ATM*	*MLH1*	*RAD50*	*AKT2*	*FLT3*	*ALK*	*FGFR3*	*PDGFRA*
AKT3	*FGFR1*	*KRAS*	*PIK3CA*	*ATR*	*MRE11*	*RAD51*	*AKT3*	*IGF1R*	*AR*	*FGR*	*PDGFRB*
ALK	*FGFR2*	*MAGOH*	*PPP2R1A*	*ATRX*	*MSH6*	*RAD51B*	*ALK*	*KIT*	*AXL*	*FLT3*	*PIK3CA*
AR	*FGFR3*	*MAP2K1*	*PTPN11*	*BAP1*	*MSH2*	*RAD51C*	*AXL*	*KRAS*	*BRCA1*	*JAK2*	*PRKACA*
ARAF	*FGFR4*	*MAP2K2*	*RAC1*	*BRCA1*	*NBN*	*RAD51D*	*AR*	*MDM2*	*BRCA2*	*KRAS*	*PRKACB*
AXL	*FLT3*	*MAP2K4*	*RAF1*	*BRCA2*	*NF1*	*RNF43*	*BRAF*	*MDM4*	*BRAF*	*MDM4*	*PTEN*
BRAF	*FOXL2*	*MAPK1*	*RET*	*CDK12*	*NF2*	*RB1*	*CCND1*	*MET*	*CDKN2A*	*MET*	*PPARG*
BTK	*GATA2*	*MAX*	*RHEB*	*CDKN1B*	*NOTCH1*	*SETD2*	*CCND2*	*MYC*	*EGFR*	*MYB*	*RAD51B*
CBL	*GNA11*	*MDM4*	*RHOA*	*CDKN2A*	*NOTCH2*	*SLX4*	*CCND3*	*MYCL*	*ERBB2*	*MYBL1*	*RAF1*
CCND1	*GNAQ*	*MED12*	*ROS1*	*CDKN2B*	*NOTCH3*	*SMARCA4*	*CCNE1*	*MYCN*	*ERBB4*	*NF1*	*RB1*
CDK4	*GNAS*	*MET*	*SF3B1*	*CHEK1*	*PALB2*	*SMARCB1*	*CDK2*	*NTRK1*	*ERG*	*NOTCH1*	*RELA*
CDK6	*H3F3A*	*MTOR*	*SMAD4*	*CREBBP*	*PIK3R1*	*STK11*	*CDK4*	*NTRK2*	*ESR1*	*NOTCH4*	*RET*
CHEK2	*HIST1H3B*	*MYC*	*SMO*	*FANCA*	*PMS2*	*TP53*	*CDK6*	*NTRK3*	*ETV1*	*NRG1*	*ROS1*
CSF1R	*HNF1A*	*MYCN*	*SPOP*	*FANCD2*	*POLE*	*TSC1*	*EGFR*	*PDGFRA*	*ETV4*	*NTRK1*	*RSPO2*
CTNNB1	*HRAS*	*MYD88*	*SRC*	*FANCI*	*PTCH1*	*TSC2*	*ERBB2*	*PDGFRB*	*ETV5*	*NTRK2*	*RSPO3*
DDR2	*IDH1*	*NFE2L2*	*STAT3*				*ESR1*	*PIK3CB*	*FGFR1*	*NTRK3*	*TERT*
EGFR	*IDH2*	*NRAS*	*TERT*				*FGF19*	*PIK3CA*			
ERBB2	*JAK1*	*NTRK1*	*TOP1*				*FGF3*	*PPARG*			
ERBB3	*JAK2*	*NTRK2*	*U2AF1*				*FGFR1*	*RICTOR*			
ERBB4	*JAK3*	*NTRK3*	*XPO1*				*FGFR2*	*TERT*			
ERCC2	*KDR*	*PDGFRA*					*FGFR3*				

© 2023 García-Pardo M et al. *JAMA Network Open.*

1C

Gene Region Content (with Target) covered by TruSight Tumor 15 with potential disease states					
**AKT1**	Exon 3* ; E17K<br>Breast	**GNA11**	Exon 5*;<br>Q209L<br>Melanoma	**NRAS**	Exons 2* ,<br>3* (partial), 4<br>Codons 12, 13, 59,<br>61, 117, 146<br>Colon
**BRAF**	Exon 15* (partial);<br>V600E/K/R/M<br>Melanoma, Colon, Lung	**GNAQ**	Exon 5* (partial);<br>Q209L<br>Melanoma	**PDGFRA**	Exons 12, 14, 18<br>Gastric, Melanoma
**EGFR**	Focal Amplification, Exons<br>12* (partial), 18, 19, 20;<br>G719A, G719X; Exon 21<br>(L858R), L861Q, S7681,<br>T790M<br>Lung	**KIT**	Exons 8, 9, 10, 11,<br>13, 14, 17, 18<br>Gastric, Melanoma	**PIK3CA**	Exons 9, 20<br>Lung, Breast,<br>Prostate
**ERBB2**	Focal Amplification,<br>p.E770_A771insAYVM<br>Exons 14* (partial), 17, 18,<br>19, 20* (partial), 21* (partial),<br>24, 26<br>Breast, Lung	**KRAS**	Exon 2* (partial), 3*<br>(partial), 4<br>Colon, Gastric, Lung	**RET**	Exon 16 (M918T)<br>Lung
**FOXL2**	Exon 1* (partial); C134W<br>Ovary	**MET**	Focal Amplification<br>Lung, Colon, Gastric	**TP53**	Full coding sequence<br>Lung, Melanoma,<br>Ovary, Colon

* = Coverage of these exons is only partial and targets specific hotspots.

© 2023 García-Pardo M et al. *JAMA Network Open*.

eTable 1. Tissue Biopsy and Molecular Testing Methods Performed

Characteristics		ACCELERATE COHORT N=150 N (%)	REFERENCE COHORT N=89 N (%)
**Tissue biopsy method**	EBUS TBNA	72 (48.0)	52 (58.4)
	CT-guided	47 (31.3)	22 (24.7)
	Thoracentesis	13 (8.7)	9 (10.1)
	Lymph node FNA	7 (4.7)	5 (5.6)
	Brain surgery	2 (1.3)	1 (1.1)
	Not biopsied	5 (3.3)	0
	Other[a]	4 (2.7)	0
**Tissue molecular profiling method**	161 gene tissue-panel	74 (82.2)	0
	15-gene tissue panel + IHC	0	63 (70.8)
	Single gene PCR testing + IHC	9 (10.0)	23 (25.8)
	Insufficient tissue	7 (7.8)	3 (3.3)
	NA[b]	60	0

[a]Other included: wedge resection (n=2), biopsy of skin lesion (n=1), pericardiocentesis (n=1)
[b]NA: not assessed. Tissue molecular profiling was only performed in patients with stage III-IV non-squamous NSCLC

Abbreviations: EBUS TBNA= endobronchial ultrasound-guided transbronchial needle aspiration; CT= computed tomography; FNA= fine needle aspiration; PCR= polymerase chain reaction; IHC= immunohistochemistry

© 2023 García-Pardo M et al. *JAMA Network Open.*

eFigure 2. Molecular Alterations in Plasma, Tissue or Both. 2A. ACCELERATE COHORT (n=53/90). 2B. In patients with successful tissue NGS (n=47/90).

2A

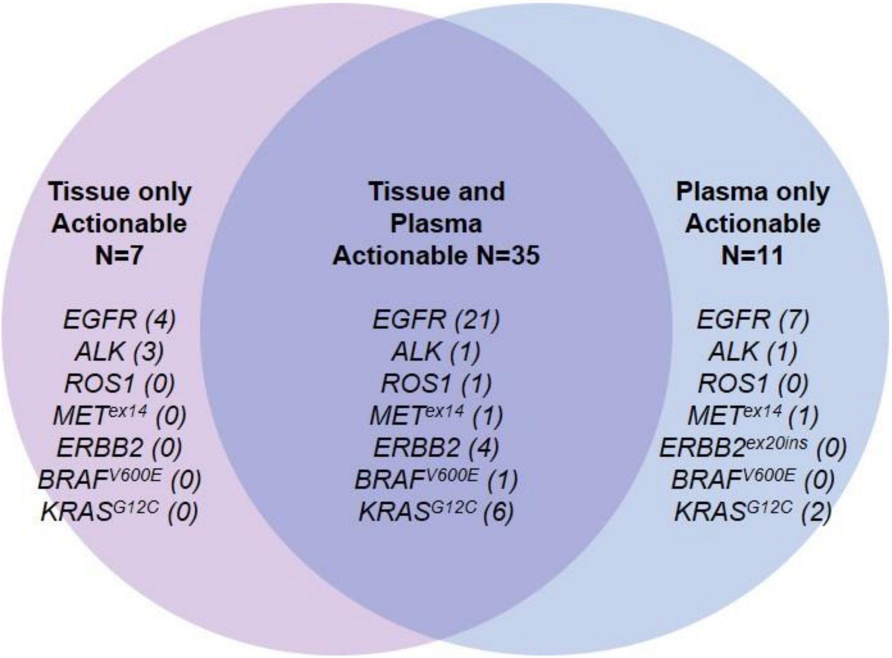

EGFR included: EGFR exon 19 del, EGFR L858R, EGFR exon 18 and EGFR exon 20 mutations

2B

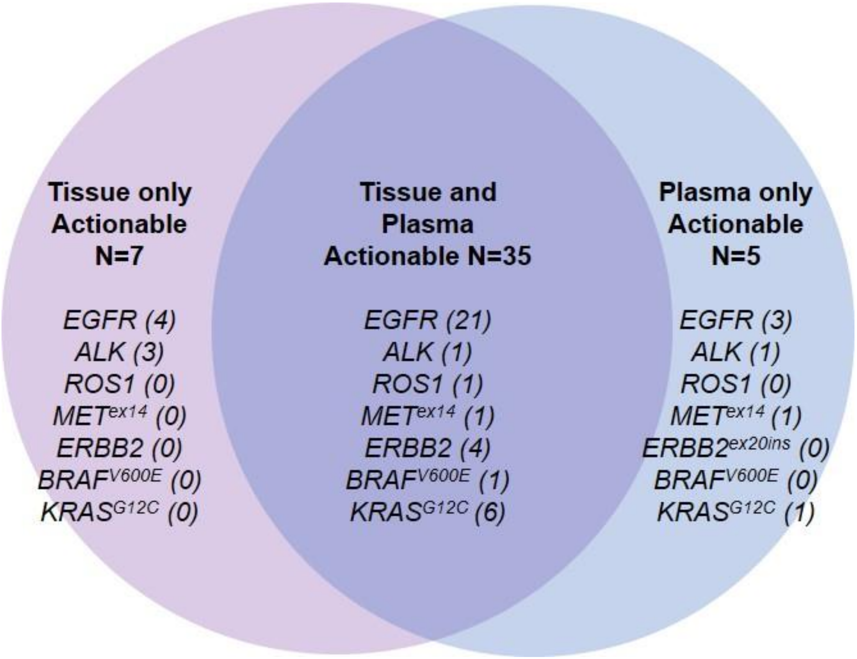

EGFR included: EGFR exon 19 del, EGFR L858R, EGFR exon 18 and EGFR exon 20 mutations

© 2023 García-Pardo M et al. *JAMA Network Open.*

eTable 2. Comparison of Tissue Versus ctDNA Results for the Guideline-Recommended Biomarkers in Advanced Non-Squamous NSCLC in the ACCELERATE Cohort (n=90)

Molecular alteration	ctDNA	Tissue +	Tissue -	Tissue NA[b]	Total	Sensitivity/ Concordance
EGFR ex19del / L858R/ atypical[a] (n=29)	ctDNA+	18	3	4	25	Sensitivity: 86.2%
	ctDNA-	0	48	3	51	
	ctDNA not detected	4	10	0	14	Concordance: 91.6%
	Total	22	61	7	90	
EGFR ex20ins (n=3)	ctDNA+	3	0	0	3	Sensitivity: 100%
	ctDNA-	0	66	7	73	
	ctDNA not detected	0	14	0	14	Concordance: 100%
	Total	3	80	7	90	
ALK fusion (n=5)	ctDNA+	1	0	1	2	Sensitivity: 40%
	ctDNA-	1	67	6	74	
	ctDNA not detected	2	12	0	14	Concordance: 96.3%
	Total	4	79	7	90	
ROS1 fusion (n=1)	ctDNA+	1	0	0	1	Sensitivity: 100%
	ctDNA-	0	68	7	75	
	ctDNA not detected	0	14	0	14	Concordance: 100%
	Total	1	82	7	90	
METex14 skip (n=2)	ctDNA+	1	1	0	2	Sensitivity: 100%
	ctDNA-	0	58	16	74	
	ctDNA not detected	0	14	0	14	Concordance: 98.6%
	Total	1	73	16	90	
ERBB2 ex20ins (n=4)	ctDNA+	4	0	0	4	Sensitivity: 100%
	ctDNA-	0	56	16	72	
	ctDNA not detected	0	14	0	14	Concordance: 100%
	Total	4	70	16	90	
BRAF V600F (n=1)	ctDNA+	1	0	0	1	Sensitivity: 100%
	ctDNA-	0	59	16	75	
	ctDNA not detected	0	14	0	14	Concordance: 100%
	Total	1	73	16	90	
KRAS G12C (n=8)	ctDNA+	6	1	1	8	Sensitivity: 100%
	ctDNA-	0	53	15	68	
	ctDNA not detected	0	14	0	14	Concordance: 98.6%
	Total	6	68	16	90	

[a] EGFR L858R (n=18), EGFR exon 19 deletion (n=10) , EGFR L861Q (n=1)
[b]Tissue NA: molecular testing not completed. N=7 for EGFR, ALK, ROS1 (single gene PCR for EGFR and immunohistochemistry for ALK and ROS1), N=16 for the rest of actionable alterations

© 2023 García-Pardo M et al. *JAMA Network Open.*

eTable 3. Variants Detected Using ctDNA Testing in Patients Other Cancer Diagnoses in ACCELERATE (n=18)

Primary cancer	Variants detected in plasma
Breast cancer	*No ctDNA detected*
Breast cancer	*ESR1*
Carcinoma of unknown primary	*KRAS, CTNNB1, PIK3CA*
Diffuse large B-cell lymphoma	*MAP2K1, TP53*
Diffuse large B-cell lymphoma	*TP53*
Diffuse large B-cell lymphoma	*No ctDNA detected*
Diffuse large B-cell lymphoma	*GNAS*
Endometrial adenocarcinoma	*TP53, IDH1*
Gastrointestinal malignancy	*TP53*
Gastrointestinal neuroendocrine carcinoma	*ERBB2* amplification
Gastrointestinal stromal tumor	*No ctDNA detected*
Hodgkin's lymphoma	*No ctDNA detected*
Melanoma	*No ctDNA detected*
Mesothelioma	*No ctDNA detected*
Plasmacytoma	*TP53, BRAF nonV600E*
Prostate adenocarcinoma	*U2AF1*
Prostate adenocarcinoma	*No ctDNA detected*
Uterine leiomyosarcoma	*TP53*

© 2023 García-Pardo M et al. *JAMA Network Open.*
