# Supplementary material for: Association of Circulating Tumor DNA Testing Before Tissue Diagnosis With Time to Treatment Among Patients With Suspected Advanced Lung Cancer: The ACCELERATE Nonrandomized Clinical Trial
Source: JAMA Netw Open. 2023 Jul 25;6(7):e2325332. doi: 10.1001/jamanetworkopen.2023.25332 (PMC10369925; doi:10.1001/jamanetworkopen.2023.25332)
Supplement: Supplement 3. — Data Sharing Statement [file jamanetwopen-e2325332-s003.pdf]

Data Sharing Statement

García-Pardo. Association of Circulating Tumor DNA Testing Before Tissue Diagnosis With Time to Treatment Among Patients With Suspected Advanced Lung Cancer. *JAMA Netw Open*. Published July 25, 2023. doi:10.1001/jamanetworkopen.2023.25332

Data
Data available: Yes
Data types: Deidentified participant data, Data dictionary
How to access data: Request to corresponding author: natasha.leighl@uhn.ca
When available: With publication

Supporting Documents
Document types: Informed consent form
How to access documents: Request to corresponding author: natasha.leighl@uhn.ca
When available: With publication

Additional Information
Who can access the data: Researchers whose proposed use of the data has been approved
Types of analyses: For a specified purpose
Mechanisms of data availability: With investigator support,and after approval of a proposal, or with a signed data access agreement
